# Effects of psychological treatments on functioning in people with Schizophrenia: a systematic review and meta-analysis of randomized controlled trials

**DOI:** 10.1007/s00406-022-01526-1

**Published:** 2022-12-08

**Authors:** Irene Bighelli, Sofia Wallis, Cornelia Reitmeir, Felicitas Schwermann, Nurul Husna Salahuddin, Stefan Leucht

**Affiliations:** Department of Psychiatry and Psychotherapy, School of Medicine, Klinikum Rechts Der Isar, Technical University of Munich, Ismaningerstraße 22, 81675 Munich, Germany

**Keywords:** Schizophrenia, Psychological interventions, Meta-analysis, Systematic review, Functioning

## Abstract

Functioning is recognized as a key treatment goal in alleviating the burden of schizophrenia. Psychological interventions can play an important role in improving functioning in this population, but the evidence on their efficacy is limited. We therefore aimed to evaluate the effect of psychological interventions in functioning for patients with schizophrenia. To conduct this systematic review and meta-analysis, we searched for published and unpublished randomized controlled trials (RCTs) in EMBASE, MEDLINE, PsycINFO, BIOSIS, Cochrane Library, WHO International Clinical Trials Registry Platform (ICTRP), ClinicalTrials.gov and the Study register of the Cochrane Schizophrenia Group. The outcome functioning was measured with validated scales. We performed random-effects pairwise meta-analysis to calculate standardized mean differences (SMDs) with 95% confidence intervals (CIs). We included 58 RCTs (5048 participants). Psychological interventions analyzed together (SMD =  – 0.37, 95% CI  – 0.49 to  – 0.25), cognitive behavioral therapy (30 RCTs, SMD =  – 0.26, 95% CI  – 0.39 to  – 0.12), and third wave cognitive-behavioral therapies (15 RCTs, SMD =  – 0.60, 95% CI  – 0.83 to  – 0.37) were superior to control in improving functioning, while creative therapies (8 RCTs, SMD = 0.01, 95% CI  – 0.38 to 0.39), integrated therapies (4 RCTs, SMD =  – 0.21, 95% CI  – 1.20 to 0.78) and other therapies (4 RCTs, SMD =  – 0.74, 95% CI  – 1.52 to 0.04) did not show a benefit. Psychological interventions, in particular cognitive behavioral therapy and third wave cognitive behavioral therapies, have shown a therapeutic effect on functioning. The confidence in the estimate was evaluated as very low due to risk of bias, heterogeneity and possible publication bias.

## Introduction

Schizophrenia is a severe mental disorder with relevant consequences for the individual and society, being ranked as one of the most debilitating disorders worldwide [2]. The disease burden for patients, relatives and society is dramatic [3, 4].

Since the first symptoms of schizophrenia typically appear in the age between 20 and 30 years, this has big impact on the life-perspectives of the young adult patients, who often do not complete their education, have difficulties in finding an occupation as well as to form relationships [1]. It is estimated that 80–90% of patients are unemployed [5]. These high rates of loss of productivity and unemployment lead to high costs for the society; with estimated total costs of more than 93 billion Euros per year, schizophrenia is among the most expensive illnesses in the EU [4].

To support patients and their families to face such challenges, it is important to address not only the symptoms of the disorder, but also the functioning of the individuals and their ability to be active members of the society. The concept of functioning is not limited to employment and economical contribution, but includes social behavior, participation and activities of daily living and self-care [6]. These aspects are included in most of the rating scales to measure functioning such as Global Assessment of Functioning (GAF) [7], Personal and Social Performance scale (PSP) [8], or the Social Functioning Scale (SFS) [9].

The importance of functioning as a therapeutic goal in schizophrenia is recognized in the scientific community [10], as well as explicitly expressed in clinical guidelines from National Institute for Care and Health Excellence (NICE) [11], Scottish Intercollegiate Guidelines Network (SIGN) [12], German Association for Psychiatry, Psychotherapy and Psychosomatics (DGPPN) [13] and other national and international guidelines.

Nevertheless, the evidence on the effects of psychological interventions on functioning in schizophrenia is very limited, and mostly focused on cognitive behavioral therapy (CBT). A network meta-analysis investigating psychological interventions in the acute phase of schizophrenia found that, on 53 included studies, 40 were focused on CBT, and only 20 had measured functioning [14]. Laws et al. conducted a meta-analysis investigating the effects of CBT on functioning, distress and quality of life [15]. Based on 25 RCTs, they found an SMD of 0.25 (95% CI 0.14–0.33) for CBT compared to control conditions [treatment as usual (TAU) or other psychological interventions]. Two Cochrane reviews by Jones et al. investigated the effects of CBT compared to TAU and compared to other psychosocial interventions and considered functioning among other outcomes. However, they provided effects for each rating scale separately and for different time points separately, resulting in analyses that include very few studies each and do not inform on the general picture [10, 16].

The evidence on other psychological interventions such as creative therapies is limited to Cochrane reviews that investigated their effect in many outcomes, but present only scattered data, separating data measured with different rating scales and at different time points [17, 18].

Randomized controlled trials have been conducted investigating other therapeutic approaches, such as third-wave cognitive therapies. After a first wave of strictly behavioral approaches, and a second characterized by the implementation of a cognitive model, the third wave of cognitive behavioral therapies includes interventions in which an emphasis is put on metacognition and how the patient relates to thoughts and emotions, such as acceptance and commitment therapy (ACT), mindfulness-based treatments and metacognitive training [19]. Integrated approaches, combining multiple fundamentally different therapeutic strategies, have been also developed and investigated [20–22].

A network meta-analysis investigated the effects of different psychological interventions in patients with schizophrenia, but was focused on patients in the acute phase, which presented positive symptoms [14]. When investigating functioning, it is important to consider also chronic patients and patients with predominant or prominent negative symptoms; in the present analysis, we included all subgroups of patients with schizophrenia.

Specific therapeutic approaches have shown different effects in patients with schizophrenia, so that it is meaningful to investigate them separately [14, 23]. On the other side, an overall picture about the efficacy of psychological interventions is missing from the literature.

The aim of the present systematic review and meta-analysis of randomized clinical trials is to provide a comprehensive overview about the efficacy of psychological interventions in improving functioning in patients with schizophrenia regardless of the comparator, time point and rating scale used. In this way, we want to answer the research question: are psychological interventions efficacious for improving functioning in patients with schizophrenia?

## Methods

### Study design and inclusion criteria

The methods of the present work were adapted from the protocol, which was registered in PROSPERO with the number CRD42017067795 and published in a peer-reviewed journal [24]. The methods have been developed according to the PRISMA statement [25]. We included studies conducted in adults with a diagnosis of schizophrenia, schizophreniform or schizoaffective disorder, with no restrictions on setting, gender or ethnicity. We excluded studies that, based on their inclusion criteria, recruited only patients with concomitant somatic or psychiatric comorbidity, or only patients with first episode psychosis. Studies were included if at least 80% of the participants had schizophrenia or related disorders (schizoaffective disorder, schizophreniform disorder, delusional disorder or non-affective psychotic disorder). We included studies regardless of the diagnostic criteria used.

Studies investigating psychological interventions were included. We considered for inclusion the interventions described in the list of psychological therapies of the Cochrane Common Mental Disorders Group (CCMD) (formerly Cochrane Collaboration Depression, Anxiety and Neurosis Group [CCDAN]) [26], such as cognitive behavioral therapy, acceptance and commitment therapy, mindfulness, art therapy and music therapy. Psychosocial and community interventions such as case management or assertive community treatment were not included, as well as family interventions. The psychological intervention was usually provided in addition to the standard care, which typically includes medication with antipsychotics [27]. We accepted as comparator another psychological intervention, inactive control, defined as interventions intended to control for non-specific aspects of the therapy (for example activity groups, befriending), treatment as usual (TAU) and waiting list.

Studies were included in the analysis if they provided data for functioning measured with a validated rating scale, such as the Global Assessment of Functioning (GAF) scale or the Social Functioning Scale (SFS) [9, 28].

### Search strategy

We searched EMBASE, MEDLINE, PsycINFO, PubMed, BIOSIS, Cochrane Library, World Health Organization’s International Clinical Trials Registry Platform and ClinicalTrials.gov for RCTs published up to January 2020 and the Study register of the Cochrane Schizophrenia Group from January 2020 up to September 2021, investigating the efficacy of psychological interventions in people with schizophrenia [29]. No time limit on how old the articles could be and no language restrictions were applied (Table 1).

### Screening and data extraction

Two reviewers among IB, SW, CR and FS screened independently all abstracts (first phase) and full texts (second phase) identified in the search for eligibility. Results of the update search from January 2018 to September 2021 were screened by IB; NHS independently re-inspected 25% of these results, to ensure reliability of selection. Disagreements were resolved by discussion, and in case of doubt, the full paper was retrieved for further inspection. Two of IB, SW, CR, FS and NHS extracted relevant data independently in a Microsoft Access database explicitly created for this study and assessed the different domains of risk of bias using the Cochrane Risk of Bias tool [30]. We also rated an overall risk of bias for each study, following the approach described by Furukawa et al. [31]. Disagreements were resolved by discussion, by involving the senior author and, in case of need, by asking the study authors. Authors of the studies were contacted via e-mail and asked if they could provide additional data relevant for the analysis.

### Data analysis

We performed random-effects pairwise meta-analyses using Review Manager version 5.3 and R Studio version 1.3.959, package meta [32, 33]. We calculated standardized mean differences (SMDs) and 95% confidence intervals (CIs). We planned different levels of analysis: (i) all psychological interventions compared to all control conditions (primary analysis); (ii) groups of psychological interventions compared to control conditions (e.g., third wave cognitive behavioral therapy, creative therapies); (iii) specific psychological interventions considered separately. The decision which studies to consider for each treatment comparison was made by two independent reviewers and then discussed, not solely based on the name the study authors gave to the intervention, but based on the description, they provided about the treatment and control conditions (Table 3).

**Table 1:** Search strategy for PsycINFO. (Created with Microsoft Office)

Search strategy for PsycINFO
1 exp Schizophrenia/
2 exp psychosis/
3 schizo$.mp
4 or/1–3
5 exp psychotherapy/ or exp Behavior Therapy/ or exp Cognitive Therapy/ or exp PSYCHOANALYSIS/ or exp psychotherapeutic counseling/ or hypnosis/ or free association/
6 (abreaction or "acceptance and commitment therapy" or acting out or adlerian or analytical psychotherap$ or anger control or anger management or animal therap$ or art therap$ or assertive$ training or attention training technique or autogenic training or autosuggestion or aversion therap$ or balint group or befriending or behavio?r contracting or behavio?r modification or behavio?r regulation or behavio?r therap$ or bibliotherap$ or biofeedback or body psychotherap$ or brief psychotherap$ or caregiver support or cbt or client cent$ or cognitive behavio?r$ or cognitive intervention$ or cognitive rehabilit$ or cognitive remediation or cognitive technique$ or cognitive therap$ or cognitive treatment$ or colo?r therap$ or compassionate mind training or conjoint therap$ or contingency management or conversational therap$ or conversion therap$ or coping skills or counsel?ing or countertransference or couples therap$ or covert sensitization or crisis intervention or dance therap$ or dialectic$ or eclectic or emotion$ focus$ or emotional freedom technique or encounter group therap$ or existential therap$ or experiential psychotherap$ or exposure therap$ or expressive psychotherap$ or eye movement desensiti?ation or family intervention$ or family therap$ or feminist therap$ or free association or freudian or geriatric psychotherap$ or gestalt therap$ or griefwork or group intervention$ or group psychotherap$ or group therap$ or guided image$ or holistic psychotherap$ or humanistic psychotherap$ or hypnosis or hypnotherap$ or hypnoti?zability or imagery or implosive therap$ or individual psychotherap$ or insight therap$ or integrated psychological therapy or integrative psychotherap$ or integrative therap$ or interpersonal or jungian or kleinian or logotherap$ or marathon group therap$ or marital therap$ or meditation or mental healing or metacognitive therap$ or metacognitive training or milieu therap$ or mindfulness or morita therap$ or multimodal or music therap$ or narrative therap$ or nondirective therap$ or object relations or person cent$ therap$ or personal construct therap$ or persuasion therap$ or pet therap$ or play therap$ or primal therap$ or problem solving or psychoanaly$ or psychodrama or psychodynamic or psychoeducat$ or psychologic$ or psychological therap$ or psychosocial treatment or psychotherap$ or psychotherapeutic counsel$ or psychotherapeutic processes or psychotherapeutic training or psychotherapeutic treatment$ or rational emotive or reality therap$ or reciprocal inhibition or rehabilitat$ or relationship therap$ or relaxation or reminiscence therap$ or rogerian or role play$ or self analys$ or self esteem or sensitivity training or sex therap$ or sleep phase chronotherap$ or social skills education or social skills training or socioenvironmental therap$ or sociotherap$ or solution focused or stress management or support group$ or supportive therap$ or systematic desensiti?ation or systemic therap$ or therapeutic communit$ or transactional analysis or transference or transtheoretical or validation therap$ or (dream$ adj3 analys$) or (support adj3 psycho$)).mp
7 or/5–6
8 ((singl$ or doubl$ or trebl$ or tripl$) adj (blind$ or mask$)).mp
9 (random$ adj5 (assign$ or allocat$)).mp
10 randomi$.mp
11 crossover.mp
12 or/8–11
13 4 and 7 and 12

Effect sizes are described according to Cohen, considering an effect size of 0.20 small, 0.50 moderate and 0.80 large [34].

We evaluated heterogeneity using *I*^2^, and considered heterogeneity probably not important for an *I*^2^ of up to 40%, moderate for an *I*^2^ from 30 to 60%, substantial for an I^2^ from 50 to 90% and considerable if over 75% according to the Cochrane Handbook for Systematic Reviews [35].

To explore potential sources for heterogeneity, we conducted subgroup and meta-regression analyses for the primary analysis, for the following potential effect modifiers: treatment setting (individual vs group), therapist expertise (trainee therapist allowed vs only expert therapists), treatment duration, age, percentage males, number of sessions, and baseline severity. Sensitivity analyses were conducted excluding studies that did not employ a blind outcome assessor, studies with researcher’s allegiance, studies focused on treatment resistant patients and studies with high overall risk of bias [31]. Subgroup, meta-regression and sensitivity analyses were considered only exploratory; therefore, we did not adjust for multiple hypotheses testing.

For the primary analysis, we assessed small trial effect, potentially associated with publication bias, by visual inspection of the funnel plot and by applying Egger’s test for funnel plot asymmetry [36]. The trim-and-fill method by Duval and Tweedie was used to give an estimate of the effect size after correcting for publication bias [37].

For the primary analysis, we assessed confidence in the estimate with the Grading of Recommendations Assessment, Development and Evaluation (GRADE) approach [38].

### Changes from protocol

*Participants*. For the present review, it was not required that patients have current positive symptoms. Studies focused on patients with predominant negative symptoms were not excluded.

*Interventions* with a primary aim different from positive symptoms were not excluded. The present review focuses on the *outcome* functioning. Pairwise meta-analyses were performed as *data analysis* method, and the GRADE approach was used to evaluate the confidence in the estimate [38].

## Results

### Characteristics of included studies

**Fig. 1:**
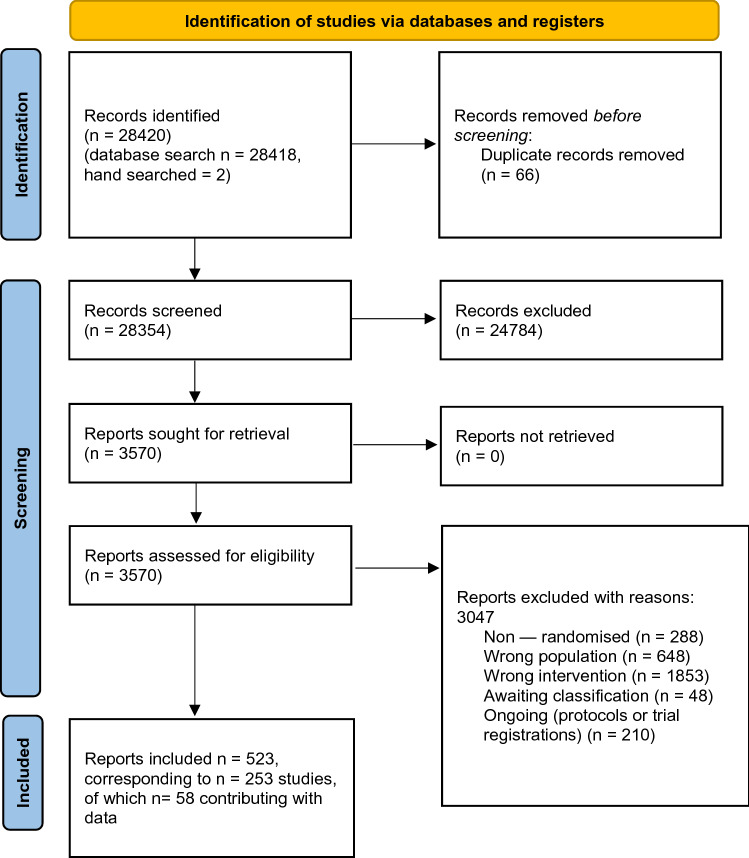
Study selection. (Created with Microsoft Office)

The search identified 28,420 records, of which 3570 were considered eligible and retrieved in full. 253 studies met the inclusion criteria, of which 58 had usable data and were included in the meta-analysis [22, 39–95]. The study selection process is illustrated in Fig. 1, and the included studies are described in Table 2.

**Table 2:** Characteristics of included studies. Arrows indicate under which term the intervention was considered for the analysis when applicable. (Adapted from [1], created with Microsoft Office)

	Overall study characteristics	Characteristics of patients
Ahuir et al [83]	Country: Spain Study treatments (number of patients): Meta Cognitive Therapy (*n* = 14), Psychoeducation (*n* = 14) Trial duration: 8 weeks Treatment setting: group Number of sessions: 8 Study design: open label Risk of bias*: moderate Functioning scale: Personal and Social Performance scale (PSP)	Diagnosis: psychotic disorder (DSM-IV) Baseline severity: Positive and Negative Syndrome Scale (PANSS) positive symptoms 9.85 Medication: 100% taking antipsychotics Only treatment resistant patients included: no
Barrowclough et al [39]	Country: UK Study treatments (number of patients): cognitive behavior therapy (CBT) (*n* = 57), treatment as usual (TAU) (*n* = 56) Trial duration: 26 weeks Treatment setting: group Therapist expertise: experts only Number of sessions: 10.4 Study design: single blind Risk of bias: moderate Functioning scale: Global Assessment of Functioning (GAF)	Diagnosis: schizophrenia or schizoaffective disorder (DSM-IV) Gender: 82 (73%) men, 31 women (27%) Mean age: 38.83 years Baseline severity: Positive and Negative Syndrome Scale (PANSS) total score: 63.8, positive symptoms 17.4, negative symptoms 14.1; Duration of illness: 13.67 years Only treatment resistant patients included: no
Bradshaw [40]	Country: not available (author’s affiliation in the USA) Study treatments (number of patients): CBT (n = 12), day treatment program (*n* = 12) Trial duration: 156 weeks Treatment setting: individual Number of sessions: not available Study design: single blind Risk of bias: high Functioning scale: Role Functioning Scale	Diagnosis: schizophrenia (DSM-IV) Setting: outpatients Mean age: 32 years Duration of illness: 11 years Medication: 100% taking antipsychotics Only treatment resistant patients included: no
Bozzatello et al [84]	Country: Italy Study treatments (number of patients): Art therapy (*n* = 30), Befriending (*n* = 32) Trial duration: 24 weeks Treatment setting: group Number of sessions: 24 Study design: open label Risk of bias: high Functioning scale: Global Assessment of Functioning (GAF) & Personal and Social Performance scale (PSP)	Diagnosis: schizophrenia spectrum disorder (DSM-V) Setting: outpatients Gender: 29 men, 25 women Mean age: 46.9 years Duration of illness: 18.8 years Medication: 100% taking antipsychotics Only treatment resistant patients included: no
Cather et al [41]	Country: USA Study treatments (number of patients): CBT (*n* = 16), psychoeducation (n = 14) Trial duration: 16 weeks Treatment setting: individual Therapist expertise: experts only Number of sessions: 15 Study design: single blind Risk of bias: high Functioning scale: Social Functioning Scale (SFS)	Diagnosis: schizophrenia or schizoaffective disorder, depressed type (DSM-IV) Setting: outpatients Gender: 17 (57%) men, 13 (43%) women Mean age: 40.4 years Baseline severity: PANSS total score 51.1, positive symptoms factor 13.53, negative symptoms factor 14.32; Duration of illness: 18 years Medication: 100% of CBT arm taking antipsychotics Only treatment resistant patients included: no
Chadwick et al [42]	Country: UK Study treatments (number of patients): mindfulness (*n* = 11), wait-list (*n* = 11) Trial duration: 10 weeks Treatment setting: group Therapist expertise: experts only Number of sessions: 10 Study design: open label Risk of bias: high Functioning scale: Clinical Outcomes in Routine Evaluation	Diagnosis: psychotic disorder (criteria not available) Mean age: 41.6 years Duration of illness: 17.7 years Medication: 100% taking antipsychotics Only treatment resistant patients included: no
Chien and Lee [43]	Country: Hongkong Study treatments (number of patients): mindfulness-based psychoeducation (*n* = 48), TAU (*n* = 48) Trial duration: 36 weeks Treatment setting: group Number of sessions: 12 Study design: single blind Risk of bias: high Functioning scale: Specific Levels of Functioning (SLOF)	Diagnosis: schizophrenia (DSM-IV) Setting: outpatients Gender: 53 (55%) men, 43 (45%) women Mean age: 25.9 years Baseline severity: Brief Psychiatric Rating Scale (BPRS) total score 63.35 Duration of illness: 3.1 years Medication: 84.375% taking antipsychotics Only treatment resistant patients included: no
Chien and Thompson [44]	Country: Hongkong Study treatments (number of patients): mindfulness-based psychoeducation (*n* = 36), TAU (*n* = 35), psychoeducation (*n* = 36) Trial duration: 27 weeks Treatment setting: group Number of sessions: 12 Study design: single blind Risk of bias: high Functioning scale: Specific Levels of Functioning (SLOF)	Diagnosis: schizophrenia (DSM-IV) Setting: outpatients Gender: 61 (57%) men, 46 (43%) women Mean age: 25.63 years Baseline severity: BPRS-18 total score 31.40 Duration of illness: 2.6 years Medication: 87.85% taking antipsychotics Only treatment resistant patients included: no
Chien et al [45]	Country: Hongkong Study treatments (number of patients): mindfulness-based psychoeducation (*n* = 114), TAU (*n* = 114), psychoeducation (*n* = 114) Trial duration: 24 weeks Treatment setting: group Therapist expertise: experts only Number of sessions: 12 Study design: single blind Risk of bias: moderate Functioning scale: Specific Levels of Functioning (SLOF)	Diagnosis: schizophrenia and other psychotic disorders (DSM-IV) Setting: outpatients Gender: 216 (63%) men, 126 (37%) women Mean age: 25.63 years Baseline severity: PANSS total score 80.77, positive symptoms 20.23, negative symptoms 19.83 Duration of illness: 2.6 years Medication: 89.77% taking antipsychotics Only treatment resistant patients included: no
Chien et al [85]	Country: China & Hong Kong Study treatments (number of patients): Mindfulness-based Psychoeducation Group Programme (MPGP) (*n* = 60), Conventional Psychoeducation Group Programme (CPGP) (*n* = 60), TAU (*n* = 60) Trial duration: 24 weeks Treatment setting: group Therapist expertise: experts only Number of sessions:12 Study design: single blind Risk of bias: moderate Functioning scale: Specific Levels of Functioning (SLOF)	Diagnosis: Schizophrenia or its subtypes according to DSM-IV-TR Setting: outpatients Gender: 100 men, 80 women Mean age: 25–28 years Baseline severity: PANSS total score: 94.73, positive symptoms 27.67, negative symptoms 23.6 Duration of illness: 2.1–2.5 years Medication: 51.6% taking first generation antipsychotics, 28.3% taking second generation antipsychotics Only treatment resistant patients included: no
Crawford et al [46]	Country: UK Study treatments (number of patients): art therapy (*n* = 140), activity group (*n* = 140), TAU (*n* = 137) Trial duration: 52 weeks Treatment setting: group Therapist expertise: experts only Number of sessions: 52 Study design: single blind Risk of bias: moderate Functioning scale: Global Assessment of Functioning (GAF)	Diagnosis: schizophrenia (clinical diagnosis) Setting: outpatients Gender: 279 (67%) men, 138 (33%) women Mean age: 41 years Baseline severity: PANSS total score 74.08, positive symptoms 17.84, negative symptoms 18.63 Duration of illness: 19.33 years Medication: 96% taking antipsychotics Only treatment resistant patients included: no
De Jong et al. 2018	Country: Netherlands Study treatments (number of patients): Metacognitive reflection and insight therapy (MERIT) (*n* = 35), treatment as usual (TAU) (*n* = 35) Trial duration: 40 weeks Treatment setting: individual Therapist expertise: experts only Number of sessions: 40 Study design: single blind Risk of bias: moderate Functioning scale: Personal and Social Performance scale (PSP)	Diagnosis: Schizophrenia or schizoaffective disorder (DSM-IV-TR) Setting: NA Gender: 49 men, 21 women Mean age: 40 years Baseline severity: PANSS total score: 66.23 Duration of illness: 13.76 years Medication: 100% taking antipsychotics Only treatment resistant patients included: no
De Pinho et al. 2020	Country: Portugal Study treatments (number of patients): Meta Cognitive Training (*n* = 27), treatment as usual (TAU) (*n* = 29) Trial duration: 4 weeks Treatment setting: group Therapist expertise: NA Number of sessions: 8 Study design: single blind Risk of bias: low Functioning scale: Personal and Social Performance scale (PSP)	Diagnosis: Schizophrenia (clinical diagnosis) Setting: unclear Gender: 30 men, 26 women Mean age: 50.48 years Baseline severity: NA Duration of illness: NA Medication: 100% taking antipsychotics Only treatment resistant patients included: no
Durham et al [47]	Country: Scotland Study treatments (number of patients): CBT (*n* = 22), supportive therapy (*n* = 23), TAU (*n* = 21) Trial duration: 39 weeks Treatment setting: individual Therapist expertise: experts only Number of sessions: 20 Study design: single blind Risk of bias: high Functioning scale: Global Assessment Scale (GAS)	Diagnosis: schizophrenia, schizoaffective disorder, delusional disorder (ICD-10 and DSM-IV) Setting: inpatients and outpatients Gender: 45 (68%) men, 21 women (32%) Mean age: 36.3 years Baseline severity: PANSS total score 96.63, PSYRATS total 35.57 Duration of illness: 13 years Medication: 100% taking antipsychotics Only treatment resistant patients included: no
Farhall et al [48]	Country: Australia Study treatments (number of patients): CBT (*n* = 45), TAU (*n* = 49) Trial duration: 52 weeks Treatment setting: individual Therapist expertise: trainees allowed Number of sessions: 17.05 Study design: open label Risk of bias: high Functioning scale: Life Skills Profile	Diagnosis: schizophrenia, schizoaffective disorder, delusional disorder, or mood disorder with psychotic features (DSM-IV) Setting: outpatients Gender: 54 (59%) men, 38 (41%) women Mean age: 32.85 years Baseline severity: PANSS total score 59.31, positive symptoms 14.63; negative symptoms 14.78 Medication: 90.43% taking antipsychotics Only treatment resistant patients included: no
Fujii et al [95]	Country: Japan Study treatments (number of patients): Meta Cognitive Training (*n* = 11), Occupational Therapy (*n* = 11) Trial duration: 16 weeks Treatment setting: group Therapist expertise: NA Number of sessions: 16 Study design: open label Risk of bias: high Functioning scale: Global Assessment of Functioning (GAF)	Diagnosis: Schizophrenia according to DSM-V Setting: inpatients Gender: 10 men, 7 women Mean age: 54.25 years Baseline severity: PANSS total score 107.8, positive symptoms 25.75; negative symptoms 28.04 Medication: 100% taking antipsychotics Only treatment resistant patients included: no
Garety et al [49] (total sample)	Country: UK Study treatments (number of patients): CBT (*n* = 133), family intervention (*n* = 28), TAU (*n* = 140) Trial duration: 39 weeks Treatment setting: individual Therapist expertise: experts only Number of sessions: 14.3 (CBT), 13.9 (FI) Study design: single blind Risk of bias: moderate Functioning scale: Social and Occupational Functioning Assessment Scale (SOFAS)	Diagnosis: non-affective psychosis (ICD-10 and DSM-IV) Setting: inpatients and outpatients Gender: 211 (70%) men, 90 (30%) women Mean age: 37.54 years Baseline severity: PANSS total score 65.16, positive symptoms 18.15, negative symptoms 13.27 Duration of illness: 10.8 years Only treatment resistant patients included: no
Garety et al [49] (sample a)	Country: UK Study treatments (number of patients): CBT (*n* = 27), family intervention (*n* = 28), TAU (*n* = 28) Trial duration: 39 weeks Treatment setting: individual Therapist expertise: experts only Number of sessions: 13.9 Study design: single blind Risk of bias: moderate Functioning scale: Social and Occupational Functioning Assessment Scale (SOFAS)	Diagnosis: non-affective psychosis (DSM-IV and ICD–10) Setting: inpatients and outpatients Gender: 60 (72%) men, 23 (28%) women Mean age: 36.4 years Baseline severity: PANSS total score 67.31, positive symptoms 17.16, negative symptoms 15.58 Duration of illness: 11.57 years Only treatment resistant patients included: no
Garety et al [49] (sample b)	Country: UK Study treatments (number of patients): CBT (*n* = 106), TAU (*n* = 112) Trial duration: 39 weeks Treatment setting: individual Therapist expertise: experts only Number of sessions: 14.3 Study design: single blind Risk of bias: moderate Functioning scale: Social and Occupational Functioning Assessment Scale (SOFAS)	Diagnosis: non-affective psychosis (DSM-IV and ICD–10) Setting: inpatients and outpatients Gender: 151 (69%) men, 67 (31%) women Mean age: 38.1 years Baseline severity: PANSS total score 64.29, positive symptoms 18.51, negative symptoms 12.38 Duration of illness: 10.4 years Only treatment resistant patients included: no
Gottlieb et al [50]	Country: USA Study treatments (number of patients): CBT (*n* = 19), TAU (*n* = 18) Trial duration: 24 weeks Treatment setting: individual Number of sessions: 10 Study design: single blind Risk of bias: moderate Functioning scale: Specific Levels of Functioning	Diagnosis: schizophrenia, schizoaffective disorder, or psychosis not otherwise specified diagnosis (NA) Setting: outpatients; 23 (62%) men, 14 women (38%) Mean age: 42.04 years Baseline severity: BPRS-24 total score 54.92, Psychotic Symptom Rating Scales (PSYRATS) 53.06, BPRS negative symptoms 6.23 Medication: 100% taking antipsychotics Only treatment resistant patients included: no
Granholm et al [51]	Country: USA Study treatments (number of patients): cognitive behavioural social skills training (CBSST) (*n* = 37), TAU (*n* = 39) Trial duration: 24 weeks Treatment setting: group Therapist expertise: experts only Number of sessions: 24 Study design: single blind Risk of bias: moderate Functioning scale: Independent Living Skills Survey (ILSS)	Diagnosis: schizophrenia or schizoaffective disorder (DSM-IV) Setting: outpatients Gender: 56 (74%) men, 20 (26%) women Mean age: 53.78 years Baseline severity: PANSS total score 53.37, positive symptoms 12.73, negative symptoms 14.66 Duration of illness: 29.23 years Medication: 87.5% taking antipsychotics Only treatment resistant patients included: no
Granholm et al [52]	Country: USA Study treatments (number of patients): CBSST (*n* = 41), goal-focused supportive contact (*n* = 38) Trial duration: 36 weeks Treatment setting: group Therapist expertise: experts only Number of sessions: 30.3 (CBSST), 29.6 (goal-focused supportive contact) Study design: single blind Risk of bias: high Functioning scale: Independent Living Skills Survey (ILSS)	Diagnosis: schizophrenia or schizoaffective disorder (DSM-IV) Setting: outpatients Gender: 44 (56%) men, 35 (44%) women Mean age: 55 years Baseline severity: PANSS total score 64.63, positive symptoms 18.06 Medication: 94.94% taking antipsychotics Only treatment resistant patients included: no
Granholm et al [53]	Country: USA Study treatments (number of patients): CBSST(*n* = 73), goal-focused supportive contact (*n* = 76) Trial duration: 36 weeks Treatment setting: group Therapist expertise: experts only Number of sessions: 12.2 (CBSST), 15.6 (goal-focused supportive contact) Study design: single blind Risk of bias: high Functioning scale: Independent Living Skills Survey (ILSS)	Diagnosis: schizophrenia or schizoaffective disorder (DSM-IV) Setting: outpatients Gender: 99 (66%) men, 50 (34%) women Mean age: 41.36 years Baseline severity: PANSS total score 72.42, positive symptoms 19.81 Duration of illness: 21.35 years Medication: 97.32% taking antipsychotics Only treatment resistant patients included: no
Granholm et al [87]	Country: United States of America Study treatments (number of patients): Mobile-assisted CBSST (MA-CBSST) (*n* = 17), Cognitive-behavioral social skills training (CBSST) (*n* = 26), device contact-only (DC-only) (*n* = 14) Trial duration: 24 weeks Treatment setting: group Number of sessions: 24 Study design: single blind Risk of bias: high Functioning scale: Independent Living Skills Survey (ILSS)	Diagnosis: schizophrenia or schizoaffective disorder (DSM-IV-TR) per the Structured Clinical Interview for DSM-IV (SCID-I/P) Setting: outpatients Gender: 47 men, 10 women Mean age: 56.1 years Baseline severity: PANSS total score: 70.64, positive symptoms 18.39 Only treatment resistant patients included: no
Grant et al [54]	Country: USA Study treatments (number of patients): cognitive therapy (*n* = 31), standard treatment (*n* = 29) Treatment setting: individual Trial duration: 18 months Number of sessions: 50.5 Study design: single blind Risk of bias: moderate Functioning scale: Global Assessment Scale (GAS)	Diagnosis: schizophrenia or schizoaffective disorder (DSM-IV) Setting: outpatients Gender: 40 (67%) men, 20 (33%) women Mean age: 38.46 years Baseline severity: Scale for the Assessment of Positive Symptoms (SAPS) score 17.33 Duration of illness: 15.52 years Only treatment resistant patients included: no
Gurcan et al 2021	Country: Turkey Study treatments (number of patients): Narrative therapy (*n* = 14), movie therapy (*n* = 14) Trial duration: 14 weeks Treatment setting: group Therapist expertise: trainee therapist allowed Number of sessions: 28 Study design: single blind Risk of bias: moderate Functioning scale: Social Functioning Scale (SFS)	Diagnosis: schizophrenia or schizoaffective disorder (DSM-V) Setting: outpatients Gender: 71,4% men, 28,6% women Mean age: 40.75 years Baseline severity: PANSS total score: 74.74, positive symptoms 16.06; negative symptoms 20.31 Duration of illness: 18.17 years Medication: 100% taking antipsychotics Only treatment resistant patients included: no
Haddock et al [55]	Country: UK Study treatments (number of patients): CBT (*n* = 38), social activity therapy (*n* = 39) Trial duration: 26 weeks Treatment setting: individual Therapist expertise: experts only Number of sessions: 17 (CBT), 17.4 (social activity therapy) Study design: single blind Risk of bias: moderate Functioning scale: Global Assessment of Functioning (GAF)	Diagnosis: schizophrenia or schizoaffective disorder (DSM-VI) Setting: inpatients and outpatients Gender: 66 (86%) men, 11 (14%) women Mean age: 34.8 years Baseline severity: PANSS total score 63.81, positive symptoms 27.6, negative symptoms 13.04 Medication: 100% taking antipsychotics Only treatment resistant patients included: no
Ishikawa et al 2019	Country: Japan Study treatments (number of patients): TAU + MCT (Meta Cognitive Training) (*n* = 24), treatment as usual (TAU) (*n* = 26) Trial duration: 10 weeks Treatment setting: group Number of sessions: 10 Study design: single blind Risk of bias: moderate Functioning scale: Global Assessment of Functioning (GAF)	Diagnosis: schizophrenia, schizotypal, and delusional disorders (ICD 10) Setting: inpatients & outpatients Gender: 25 men, 25 women Mean age: 47.5 years Baseline severity: positive symptoms 23.78 Duration of illness: 21.04 years Medication: 100% taking antipsychotics Only treatment resistant patients included: no
Jenner et al [20]	Country: Netherlands Study treatments (number of patients): hallucination focused integrative treatment (*n* = 39), TAU (*n* = 39) Trial duration: 39 weeks Treatment setting: group Therapist expertise: experts only Number of sessions: 11 Study design: open label Risk of bias: high Functioning scale: Groningen Social Disabilities Schedule	Diagnosis: non-affective psychosis, including schizophrenia, schizoaffective or psychotic disorder not otherwise specified (DSM-IV) Setting: outpatients Gender: 41 (54%) men, 35 (46%) women Mean age: 36.35 years Baseline severity: PANSS total score 60.2, positive symptoms 16.05, negative symptoms 13.25 Only treatment resistant patients included: no
Klingberg et al [57]	Country: Germany Study treatments (number of patients): CBT (*n* = 99), cognitive remediation (*n* = 99) Trial duration: 36 weeks Treatment setting: individual Therapist expertise: trainees allowed Number of sessions: 16.6 (CBT), 13.7 (cognitive remediation) Study design: single blind Risk of bias: moderate Functioning scale: Global Assessment of Functioning (GAF)	Diagnosis: schizophrenia (DSM-IV) Setting: outpatients Gender: 87 women (43.94%), 111 men (56.06%) Mean age: 36.9 years Baseline severity: PANSS total score 59.45, positive symptoms 10.5, negative symptoms 18.55 Duration of illness: 12.5 years Medication: 100% taking antipsychotics Only treatment resistant patients included: no
Kråkvik et al [58]	Country: Norway Study treatments (number of patients): CBT (*n* = 23), wait-list (*n* = 22) Trial duration: 26 weeks Treatment setting: individual Therapist expertise: trainees allowed Number of sessions: 20 Study design: open label Risk of bias: moderate Functioning scale: Global Assessment of Functioning (GAF)	Diagnosis: schizophrenia, schizoaffective disorder, or persistent delusional disorder (ICD-10) Setting: inpatients and outpatients Gender: 29 (64%) men, 16 (36%) women Mean age: 36.36 years Baseline severity: BPRS-24 score 49.49 Duration of illness: 10.9 years Only treatment resistant patients included: no
Kuipers et al [59]	Country: UK Study treatments (number of patients): CBT and family intervention (*n* = 32), TAU (*n* = 27) Trial duration: 39 weeks Number of sessions: not available Study design: single blind Risk of bias: moderate Functioning scale: Global Assessment of Functioning (GAF)	Diagnosis: any functional psychosis (OPCRIT) Setting: outpatients Gender: 45 (76%) men, 14 (24%) women Mean age: 27.8 years Baseline severity: PANSS total score 73.11, positive symptoms 17.39, negative symptoms 16.86 Only treatment resistant patients included: no
Lee et al [60]	Country: Korea Study treatments (number of patients): group music therapy (*n* = 12), control (*n* = 12) Trial duration: 12 weeks Treatment setting: group Number of sessions: 18 Study design: not available Risk of bias: high Functioning scale: Global Assessment of Functioning (GAF)	Diagnosis: schizophrenia (DSM-IV) Setting: outpatients Gender: 25 (75%) men, 5 (25%) women Mean age: 40.55 years Baseline severity: PANSS total score 94.5, positive symptoms 21.25, negative symptoms 23.2 Only treatment resistant patients included: no
Li et al 2015	Country: China Study treatments (number of patients): CBT (*n* = 96), supportive therapy (*n* = 96) Trial duration: 24 weeks Treatment setting: individual Therapist expertise: experts only Number of sessions: 15 Study design: single blind Risk of bias: moderate Functioning scale: Personal and Social Performance scale (PSP)	Diagnosis: schizophrenia (DSM-IV) Setting: inpatients and outpatients Gender: 72 (38%) men, 120 (63%) women Mean age: 31.36 years Baseline severity: PANSS total score 72.6, positive symptoms 23.43, negative symptoms 20.4 Duration of illness: 8.21 years Medication: 100% taking antipsychotics Only treatment resistant patients included: no
Lincoln et al [62]	Country: Germany Study treatments (number of patients): CBT (*n* = 40), TAU (*n* = 40) Trial duration: 38 weeks Treatment setting: individual Therapist expertise: trainees allowed Number of sessions: 28.9 (CBT), 2 (TAU) Study design: open label Risk of bias: high Functioning scale: Global Assessment of Functioning (GAF)	Diagnosis: schizophrenia, schizoaffective disorder, delusional disorder, or brief psychotic disorder (DSM-IV) Setting: outpatients Gender: 45 (56%) men, 35 (44%) women Mean age: 33.15 years Baseline severity: PANSS total score 63.15, positive symptoms 14.95, negative symptoms 14.15 Duration of illness: 10.4 years Medication: 96.25% taking antipsychotics Only treatment resistant patients included: no
Martin et al [63]	Country: Germany Study treatments (number of patients): dance and movement therapy and body psychotherapy (*n* = 44), TAU (*n* = 24) Trial duration: 10 weeks Treatment setting: group Therapist expertise: trainees allowed Number of sessions: 20 Study design: single blind Risk of bias: high Functioning scale: Global Assessment of Functioning (GAF)	Diagnosis: schizophrenia spectrum disorder (ICD-10) Setting: outpatients Gender: 36 (53%) men, 32 (47%) women Mean age: 39.8 years Baseline severity: BPRS total score 38.16, SANS 25.03 Medication: 100% taking antipsychotics Only treatment resistant patients included: no
Matthews [64]	Country: USA Study treatments (number of patients): psychotherapy (*n* = 28), TAU (*n* = 14) Trial duration: 8 weeks Therapist expertise: experts only Number of sessions: 8 Study design: not available Risk of bias: high Functioning scale: Global Assessment of Functioning (GAF)	Diagnosis: schizophrenia (NA) Setting: outpatients Gender: 21 (50%) men, 21 (50%) women Mean age: 24.95 years Medication: 100% taking antipsychotics Only treatment resistant patients included: no
Montag et al [65]	Country: Germany Study treatments (number of patients): psychodynamic art therapy (*n* = 29), TAU (*n* = 29) Trial duration: 6 weeks Number of sessions: 12 Study design: single blind Risk of bias: high Functioning scale: Global Assessment of Functioning (GAF)	Diagnosis: schizophrenia (DSM-IV) Setting: inpatients Gender: 38 (72%) men, 15 (28%) women Mean age: 38.1 years Baseline severity: SAPS total score 60.15, Scale for the Assessment of Negative Symptoms (SANS) 45.6 Duration of illness: 12.6 years Only treatment resistant patients included: no
Morrison et al [66]	Country: UK Study treatments (number of patients): cognitive therapy (*n* = 37), TAU (*n* = 37) Trial duration: 39 weeks Therapist expertise: experts only Number of sessions: 13.3 Study design: single blind Risk of bias: moderate Functioning scale: Personal and Social Performance scale (PSP)	Diagnosis: schizophrenia, schizoaffective disorder, or delusional disorder; diagnostic uncertainty in early phases of psychosis (Early intervention for psychosis service) (ICD-10 or PANSS) Gender: 39 (53%) men, 35 (47%) women Mean age: 31.32 years Baseline severity: PANSS total score 71.76, positive symptoms 20.98, negative symptoms 14.52; Medication: 0% taking antipsychotics Only treatment resistant patients included: no
Morrison et al [90]	Country: United Kingdom Study treatments (number of patients): cognitive behavior therapy (CBT) (*n* = 242), treatment as usual (TAU) (*n* = 245) Trial duration: 39 weeks Treatment setting: individual Therapist expertise: only expert therapists Number of sessions: 26 Study design: single blind Risk of bias: moderate Functioning scale: Personal and Social Performance scale (PSP)	Diagnosis: schizophrenia, schizoaffective disorder, or delusional disorder, or criteria for an Early Intervention for Psychosis service to allow for diagnostic uncertainty in early phases (ICD-10) Setting: inpatients and outpatients Gender: 349 men, 138 women Mean age: 42.5 years Baseline severity: PANSS total score: 83.05; positive symptoms 24.95; negative symptoms 19.35 Duration of illness: 19 years Medication: 91% taking antipsychotics Only treatment resistant patients included: yes
Naeem et al [67]	Country: Canada Study treatments (number of patients): CBT (*n* = 18), TAU (*n* = 15) Trial duration: 16 weeks Treatment setting: individual Number of sessions: 14 Study design: single blind Risk of bias: low Functioning scale: (World Health Organization Disability Assessment Schedule) WHODAS 2.0	Diagnosis: schizophrenia (DSM-IV) Setting: outpatients Gender: 17 (52%) men, 16 (48%) women Mean age: 40.45 years Baseline severity: PANSS total score 50.24, positive symptoms 13.54, negative symptoms 12.18 Only treatment resistant patients included: no
Ochoa et al [68]	Country: Spain Study treatments (number of patients): metacognitive training (*n* = 65), psychoeducation (*n* = 57) Trial duration: 8 weeks Treatment setting: group Therapist expertise: experts only Number of sessions: 5.53 (metacognitive training), 4.95 (psychoeducation) Study design: single blind Risk of bias: high Functioning scale: Global Assessment of Functioning (GAF)	Diagnosis: schizophrenia, psychotic disorder not otherwise specified, delusional disorder, schizoaffective disorder, brief psychotic disorder, or schizophreniform disorder (DSM-IV-TR) Setting: outpatients Gender: 85 (70%) men, 37 (30%) women Mean age: 27.59 years Baseline severity: PANSS total score 54.33, positive symptoms 12.22, negative symptoms 14.63 Duration of illness: 2.29 years Only treatment resistant patients included: no
Ozdemir and Budak 2022	Country: Turkey Study treatments (number of patients): mindfulness-based stress reduction (MBSR) (*n* = 50), psychoeducation group (*n* = 50), control group (*n* = 56) Trial duration: 8 weeks Treatment setting: group Therapist expertise: only expert therapists Number of sessions: 8 Study design: open label Risk of bias: high Functioning scale: Functional Remission of General Schizophrenia (FROGS) scale	Diagnosis: schizophrenia (DSM-V) Setting: outpatients Gender: 106 men, 31 women Mean age: 43.77 years Duration of illness: 16.36 years Medication: 100% taking antipsychotics Only treatment resistant patients included: no
Palma et al [22]	Country: Spain Study treatments (number of patients): Cognitive-Motivational Therapy (PIPE) (*n* = 35), Routine Care (*n* = 27) Trial duration: 52 weeks Treatment setting: individual & family Therapist expertise: expert only Number of sessions: 34 Study design: single blind Risk of bias: moderate Functioning scale: Global Assessment of Functioning (GAF)	Diagnosis: initial phase of schizophrenia (DSM-IV) Setting: inpatients & outpatients Gender: 46 men, 16 women Mean age: 25.5 years Baseline severity: PANSS total score: 104, positive symptoms 38,8, negative symptoms 30,7 Duration of illness: 1 year Only treatment resistant patients included: no
Penadés et al [69]	Country: Spain Study treatments (number of patients): CBT (*n* = 20), cognitive remediation (*n* = 20) Trial duration: 16 weeks Treatment setting: individual Therapist expertise: experts only Number of sessions: 40 Study design: single blind Risk of bias: moderate Functioning scale: Life Skills Profile	Diagnosis: schizophrenia (DSM-IV) Setting: outpatients Gender: 23 men (57.5%), 17 women (42.5%) Mean age: 35.1 years Baseline severity: PANSS score 66.99, positive symptoms 11.27, negative symptoms 20.17 Duration of illness: 13.8 years Medication: 100% taking antipsychotics Only treatment resistant patients included: no
Penn et al [70]	Country: USA Study treatments: CBT (*n* = 32), supportive therapy (*n* = 33) Trial duration: 12 weeks Treatment setting: group Number of sessions: 8.3 Study design: single blind Risk of bias: low Functioning scale: Social Functioning Scale (SFS)	Diagnosis: schizophrenia or schizoaffective disorder (DSM-IV) Setting: outpatients Gender: 33 (51%) men, 32 (49%) women Mean age: 40.65 years Baseline severity: PANSS total score 61.75, positive symptoms 17.55, negative symptoms 13.9 Duration of illness: 15.4 years Only treatment resistant patients included: no
Pos et al [92]	Country: Netherlands Study treatments (number of patients): CBTsa (Social Activation) (*n* = 49), TAU (*n* = 50) Trial duration: 12 weeks Treatment setting: group & individual Therapist expertise: experts only Number of sessions: 14 Study design: single blind Risk of bias: moderate Functioning scale: Global Assessment of Functioning (GAF)	Diagnosis: schizophrenia or a related disorder with onset of their first psychotic episode 4 years prior to inclusion (DSM-IV-TR) Setting: inpatients & outpatients Gender: male 80, female 19 Mean age: 25.43 years Baseline severity: NA Duration of illness: NA Medication: 91.7% taking antipsychotics Only treatment resistant patients included: no
Pot-Kolder et al [26]	Country: Netherlands Study treatments (number of patients): virtual-reality exposure therapy for psychosis (*n* = 58), wait-list (*n* = 58) Trial duration: 12 weeks Number of sessions: 16 Study design: single blind Risk of bias: low Functioning scale: Social and Occupational Functioning Assessment Scale (SOFAS)	Diagnosis: psychotic disorder (DSM-IV) Gender: 82 (71%) men, 34 (29%) women Mean age: 38 years Duration of illness: 14.1 years Medication: 95.5% taking antipsychotics Only treatment resistant patients included: no
Richardson et al [72]	Country: UK Study treatments (number of patients): art therapy (*n* = 43), TAU (*n* = 47) Trial duration: 12 weeks Treatment setting: group Number of sessions: 12 Study design: open label Risk of bias: high Functioning scale: Social Functioning Scale (SFS)	Diagnosis: chronic schizophrenia (NA) Setting: outpatients Gender: 59 (66%) men, 31 (34%) women Mean age: 41.17 years Baseline severity: BPRS total score 15.57, SANS 8.44 Only treatment resistant patients included: no
Schrank et al [73]	Country: UK Study treatments (number of patients): group psychotherapy (*n* = 47), TAU (*n* = 47) Trial duration: 11 weeks Treatment setting: group Number of sessions: 7 Study design: open label Risk of bias: high Functioning scale: Health of the Nation Outcome Scale	Diagnosis: diagnosis of psychosis defined as schizophrenia and other psychoses including schizoaffective and delusional disorder but not depressive psychosis or psychosis due to substance misuse (clinical diagnosis) Setting: inpatients and outpatients Gender: 56 (60%) men, 38 (40%) women Mean age: 42.5 years Baseline severity: BPRS-18 total score 32.14 Duration of illness: 13.5 years Only treatment resistant patients included: no
Shawyer et al [74]	Country: Australia Study treatments (number of patients): CBT (*n* = 21), befriending (*n* = 22) Trial duration: 15 weeks Treatment setting: individual Therapist expertise: experts only Number of sessions: 14.3 (CBT), 14.4 (Befriending) Study design: single blind Risk of bias: high Functioning scale: Global Assessment of Functioning (GAF)	Diagnosis: schizophrenia or related condition (DSM-IV) Gender: 24 (56%) men, 19 (44%) women Mean age: 39.8 years Baseline severity: PANSS total score 62.89, positive symptoms 15.99, negative symptoms 14.15 Duration of illness: 14.71 years Only treatment resistant patients included: no
Shawyer et al [75]	Country: Australia Study treatments (number of patients): acceptance and commitment therapy (*n* = 49), befriending (*n* = 47) Trial duration: 13 weeks Treatment setting: individual Therapist expertise: experts only Number of sessions: 7 Study design: single blind Risk of bias: low Functioning scale: Social Functioning Scale (SFS)	Diagnosis: schizophrenia or schizoaffective disorder (DSM-IV-TR) Setting: outpatients Gender: 59 (61%) men, 37 (39%) women Mean age: 34.3 years Baseline severity: PANSS total score 78.25, positive symptoms 21.8, negative symptoms 18 Medication: 100% taking antipsychotics Only treatment resistant patients included: no
Startup et al [76]	Country: UK Study treatments (number of patients): CBT (*n* = 47), TAU (*n* = 43) Trial duration: 26 weeks Treatment setting: individual Therapist expertise: experts only Number of sessions: 12.9 Study design: open label Risk of bias: high Functioning scale: Global Assessment of Functioning (GAF)	Diagnosis: schizophrenia, schizophreniform, schizoaffective (DSM-IV) Setting: inpatients Gender: 68 (76%) men, 22 (24%) women Mean age: 30.8 years Baseline severity: BPRS-16 total score 45.75, SAPS positive symptoms 10.7, SANS negative symptoms 8.9; Duration of illness: 6.95 years Only treatment resistant patients included: no
Talwar et al [77]	Country: UK Study treatments (number of patients): music therapy (*n* = 33), TAU (*n* = 48) Trial duration: 12 weeks Treatment setting: individual Number of sessions: 8 Study design: single blind Risk of bias: low Functioning scale: Global Assessment of Functioning (GAF)	Diagnosis: schizophrenia, or schizophrenia-like psychoses (ICD-10) Setting: inpatients Gender: 60 (74%) men, 21 (26%) women Mean age: 37.36 years Baseline severity: PANSS total score 71.72, positive symptoms 16.36, negative symptoms 19.20 Only treatment resistant patients included: no
Tarrier et al [78]	Country: UK Study treatments: CBT (*n* = 25), TAU (*n* = 24) Trial duration: 16 weeks Treatment setting: individual Therapist expertise: experts only Number of sessions: 24 Study design: single blind Risk of bias: moderate Functioning scale: Global Assessment of Functioning (GAF)	Diagnosis: schizophrenia, schizophreniform disorder, schizoaffective disorder, delusional disorder or psychotic disorder not otherwise specified (DSM-IV) Gender: 31 (63%) men, 18 (37%) women Mean age: 34.9 years Baseline severity: PANSS total score 60.12, positive symptoms 15.44, negative symptoms 13.29 Medication: 100% taking antipsychotics O Only treatment resistant patients included: no
Valencia et al]. [79]	Country: Mexico Study treatments (number of patients): music therapy (*n* = 18), psychosocial therapy (*n* = 18), multiple therapies (*n* = 18) Trial duration: 26 weeks Number of sessions: 44 (MT), 44 (PST), 108 (MultipleT) Study design: single blind Risk of bias: high Functioning scale: Global Assessment of Functioning (GAF)	Diagnosis: schizophrenia (DSM-IV) Setting: outpatients Gender: 33 (77%) men, 10 (23%) women Mean age: 30.5 years Duration of illness: 8.13 years Medication: 100% taking antipsychotics Only treatment resistant patients included: yes
van der Gaag et al [80]	Country: Netherlands Study treatments (number of patients): CBT (*n* = 110), TAU (*n* = 106) Treatment setting: individual Trial duration: 26 weeks Number of sessions: 13 Study design: single blind Risk of bias: high Functioning scale: Social Functioning Scale (SFS)	Country: schizophrenia or schizoaffective disorder (DSM-IV-TR) Gender: 153 (71%) men, 63 (29%) women Mean age: 36.99 years Baseline severity: PANSS total score 69.3, PSYRATS total 31.35 Duration of illness: 10.58 years Only treatment resistant patients included: no
Wang et al [81]	Country: Hong Kong Study treatments (number of patients): mindfulness-based psychoeducation (*n* = 46), psychoeducation (*n* = 46), TAU (*n* = 46) Trial duration: 26 weeks Treatment setting: group Therapist expertise: experts only Number of sessions: 12 Study design: single blind Risk of bias: moderate Functioning scale: Specific Levels of Functioning (SLOF)	Diagnosis: schizophrenia or its subtypes (DSM-IV-TR) Setting: outpatients Gender: 72 (52%) men, 66 (48%) women Mean age: 24.3 years Baseline severity: PANSS total score 87.93, positive symptoms 26.57, negative symptoms 18.3 Duration of illness: 2.03 years Medication: 85.51% taking antipsychotics Only treatment resistant patients included: no
Wykes et al [82]	Country: UK Study treatments (number of patients): CBT (*n* = 45), TAU (*n* = 40) Trial duration: 10 weeks Treatment setting: group Therapist expertise: experts only Number of sessions: 7 Study design: open label Risk of bias: high Functioning scale: Social Behaviour Schedule	Diagnosis: schizophrenia (DSM-IV) Setting: outpatients Gender: 50 (59%) men, 35 (41%) women Mean age: 39.7 years Baseline severity: PSYRATS hallucination score 27.95 Only treatment resistant patients included: no
Yildiz et al. [93]	Country: Turkey Study treatments (number of patients): Psychosocial Skills Training (PST) (*n* = 10), Meta Cognitive Training (MCT) (*n* = 10) Trial duration: 20 weeks Treatment setting: group Therapist expertise: only expert Number of sessions: 40 Study design: single blind Risk of bias*: moderate Functioning scale: Global Assessment of Functioning (GAF)	Diagnosis: schizophrenia or schizoaffective disorder (DSM-IV) Setting: outpatients Gender: 13 men, 7 women Mean age: 35.25 years Baseline severity: PANSS total score: 81.85 Duration of illness: 13.4 years Medication: 100% taking antipsychotics Only treatment resistant patients included: no

^*^The overall risk of bias was calculated using the Cochrane risk of bias tool (Higgins et al. [30]) and the approach described by Furukawa [31]. For this evaluation, the domain “blinding of participants and personnel” was not considered as patients and therapists usually cannot be blinded in psychological interventions

The different psychological interventions investigated in the studies are described in detail in Table 3.

**Table 3:** Description of interventions. (Adapted from [1], created with Microsoft Office)

Acceptance and commitment therapy (ACT)	A manualized cognitive behavior therapy (Hayes et al. 2003, p. 79) that focuses more on the patient’s relation to distressing symptoms than on the symptoms themselves. It encourages patients to be mindful of and accept instead of try and avoid negative experiences, such as distressing voices. At the same time, it is a goal to take value-guided action to enable positive change in spite of the difficulties the patients face^1^
Activity group ( inactive control)	Activity groups are used as control groups in the trial Crawford and colleagues conducted. They aim to control for potential effects of the group setting also used in group art therapy. Lead by a group facilitator, the patients engage together in different activities varying from watching films to visiting local cafés. Psychological techniques as well as art materials are not employed^2^
Art therapy	In art therapy, patients express their inner experience spontaneously and freely in a creative process using different art materials. Then, they get the possibility to share and discuss their pictures helped by interventions of an art therapist^2,3^
Befriending ( inactive control)	A manualized treatment designed to control for the therapist’s attention and the patient’s treatment expectancy. It includes conversation about everyday topics and, if conversation is too difficult to attain, neutral activities that do not provoke fear or negative emotions. For talking about symptoms and problems, the patient is referred to the treating clinician^4^
Cognitive-behavioral social skills training ( CBT)	An intervention integrating cognitive behavioral techniques and strategies from social skills training to help patients challenge their thoughts, ask for help in an appropriate way and problem-solve, tailored to the specific needs of patients suffering from schizophrenia^5^
Cognitive behavior therapy (CBT)	A widely spread therapy approach aimed at changing thought processes and behavior. Initially, a stable therapeutic relationship is to be built. The following treatment includes identifying dysfunctional cognitive and behavioral patterns, setting distinct and reachable therapy goals together and replacing dysfunctional patterns step by step with healthier ones. CBT for psychosis focusses especially on dealing with disturbing hallucinations and delusional thoughts as well as the identification of negative belief systems and the development of healthy coping strategies^6^
Cognitive remediation	Applying the principles of errorless learning and immediate positive feedback, executive functioning, attention and memory are trained using techniques for structuring of information, verbalization and self-instruction^7^
Cognitive therapy ( CBT)	An individualized goal-directed therapy approach aiming to motivate the patient to work on realistic long- and short-term goals. Dysfunctional believes are replaced by more functional ones using cognitive and behavioral strategies. Techniques introduced and practiced during the sessions are consolidated with homework for the patient to do between the sessions^8^
Creative therapy	This term summarizes therapies that give patients the possibility to express themselves in a creative way, for example through art, music or body movement. For more detailed information about the treatments that are considered creative therapy, see their descriptions in this table
Dance and movement therapy and body psychotherapy	See movement therapy
Day treatment program ( TAU)	A psychiatric service provided for a longer period of time to persons with serious and chronic psychiatric conditions. It entails medication management and different group interventions^9^
Family intervention	An intervention that aims at improving communication and problem-solving skills in the families of schizophrenic patients. There are psychoeducational elements to enable a better understanding of the patients. On top of that, the patient and the relatives get the possibility to discuss and resolve conflicts with the help of a professional and work through difficult emotions that arise as a consequence of the disease^10^
Goal-focused supportive contact ( inactive control)	Designed to control for frequency and amount of contact to the therapist and other group members, this intervention gives patients the opportunity to formulate goals and work on them through group discussion without specific therapist guidance^11^
Hallucination focused integrative treatment	This is a combination treatment containing psychoeducational, cognitive-behavioral, coping-oriented as well as family interventions and rehabilitative elements added to antipsychotic medication. The main purpose is to cope better with hallucinations^12^
Inactive control	This term is utilized for any treatment that serves as a control condition regarding non-specific factors such as the therapist’s attention, for example “activity group”, “befriending”, “social activity therapy” or “supportive counselling” which are also described in this table^13^
Integrated therapies	Under this term, treatments that combine multiple fundamentally different therapeutic strategies, for example music therapy, family intervention or behavioral therapy, are subsumed
Metacognitive training	A structured group intervention aimed at dismantling cognitive biases that contribute to psychotic exacerbations. There are multiple modules with different specific targets such as showing the importance of collecting enough information before making assumptions, strengthening theory of mind or also handling affective symptoms^14^
Metacognitive reflection and insight therapy (MERIT) ( metacognitive training)	This therapy aims to stimulate the four metacognition elements (self-reflectivity, understanding the other’s mind, decentration, and mastery) and focuses on adjusting level of metacognition of the patient during the session rather than providing a step-by-step intervention^28^
Mindfulness	A third-wave cognitive and experiential approach aimed at enabling a different pattern of relating to psychotic experiences such as thoughts, images and hallucinations. Core element are guided meditation sessions in which patients are motivated to focus on bodily sensations and their breath and bring a gentle attention to distressing symptoms. The aim is not to eliminate distressing sensations, but to alleviate distress that is generated by dysfunctional ways of relating to them. Mindfulness meditation integrated with discussion in a cognitive frame is believed to bring about metacognitive insights that enhance the process of relating more functionally to psychotic experiences^15,16^
Mindfulness-based psychoeducation ( mindfulness)	Its aim is to increase the patient’s comprehension of schizophrenia and their illness insight as well as helping them to manage and accept their symptoms. Patients are taught to recognize and respond in a less-involved way to their emotions, cognitions and perceptions instead of seeing them as exact representations of reality^17^
Movement therapy	Movement therapy is a therapy form that aims at alleviating psychotic symptoms using body-oriented exercises. These can focus on perception of sensation on the one hand as well as on an active and expressive bodily movement on the other hand. Feeling and moving the body are used to enable the patients to develop a more embodied sense of self, a broader range of communicative behaviors and a more differentiated understanding and expression of their emotions^18^
Movie therapy ( inactive control)	A therapy that screens the 2–3-min videos on different topics showing the people interact each other and reflecting certain emotion theme such as basic emotions (fear and happiness) and complex emotions (jealousy, disappointment, etc.). This movie therapy allows the patients to follow three stages which first the video sections are watched without interruption and the group therapist will ask the patient how they interpret the scenes. In the second stage, the group therapist will pause the video sections to allow the patients to collect the social clues such as place, time, facial expressions, voice tones, etc. Finally, the patients discuss the social clues they have picked up^29^
Music therapy	An intervention that uses music to tackle psychotic symptoms. It can take place either individually or in a group setting. Usually, patients are encouraged to express themselves spontaneously by improvising on musical instruments. Therapeutic interventions can entail accompanying the patients’ music, helping them to vary the course of the music and interpreting the music together through discussion^19^
Narrative therapy	Narrative therapy is an intervention that helps patients to collect their life events, experiences, and memories, as well as understand other people’s thoughts and feelings to gain new insights and perspectives. There are four stages during the sessions. At the first stage, the patients will share their stories without interruption after the group therapist reviewed the narrative before the session begins. The second and third stage will be about responding to the narrative by asking several questions and it is also important to ask the patient’s emotions during the event and ensure that the patient feel accepted of what other people might have thought and felt. The last stage aims to gain an insight and perspective from other patients^29^
PIPE (Psychoeducation, CBT, motivational intervention)	This is a combination therapy consisting of psychoeducation, individual CBT & family cognitive-motivational therapy. The aims to treat the patient’s ideas and hallucination and to ensure the family can adapt to the illness and provide the optimal support and environment for the sake of patient’s recovery^30^
Psychotherapy not further specified	A therapy is considered not specified, if no further information is given about the specific form of psychological treatment, for example in the study undertaken by Matthews in 1981, where it was only mentioned that the patients received “psychotherapy”, but no details were given
Positive psychotherapy	Using different exercises, patients shall be encouraged to make positive experiences, enhance their personal strengths and interpersonal relationships and get a more meaningful perspective on their lives^20^
Psychodynamic therapy	Patients get the opportunity to describe the narratives of their lives. By doing so, they can make sense of the timing and nature of the illness and how it is related to strong and unbearable affects in their past personal history. Furthermore, transference phenomena in the therapeutic relationship can be described and worked through^24^
Psychoeducation	Psychoeducation is meant to teach patients about different aspects of their disease and its management. Topics vary from explanation models of disease development to the rationale for medication and different coping strategies as well as noticing and understanding warning signs for relapses^21^
Psychosocial therapy	Psychosocial therapy is an intervention based on social skills training with the aim to give patients behavioral alternatives that enrich their existing behavioral strategies. The focus lies on five areas: occupation, economical aspects and relationships with friends, partners and family^22^
Social activity therapy ( inactive control)	Social activity therapy has the goal to support patients in finding activities they like doing and taking steps to actually engage in them^23^
Standard treatment ( TAU)	See “Treatment as usual”
Supportive therapy	In supportive therapy, a safe environment is created in which patients can talk about their problems^24^. The therapists support the patients emotionally without giving symptom specific interventions. More importance is given to non-specific therapeutic factors such as empathic attitude and creating a reliable therapeutic alliance^25^
Third-wave cognitive behavioral therapy	If described as waves, the first wave of CBT consists in the strictly behavioral approach and the second is characterized by the implementation of a cognitive model. In the current third wave, an emphasis is put on metacognition and how the patient relates to thoughts and emotions. Examples for third-wave therapies are dialectical behavior therapy, acceptance and commitment therapy (ACT), mindfulness-based treatments, metacognitive therapy and several others^27^
Treatment as usual (TAU)	Patients assigned to this group get the usual psychiatric care. What it exactly entails depends on the local guidelines. Usually, patients are offered medication and regular visits to doctors and nurses to talk about current issues^13^
Virtual-reality exposure therapy for psychosis ( CBT)	A cognitive behavior therapy using a virtual-reality environment for exposure exercises for fear and paranoia provoking social situations^26^
Wait-list	If patients get assigned to the wait-list, this means that they get informed that there is a possible treatment, but they cannot yet engage in it. They can only get that specific treatment after waiting some weeks^13^

1. Pankey J, Hayes SC (2003) Acceptance and commitment therapy for psychosis. Int J Psychol Psychol Ther 3(2):311–328

2. Crawford MJ, Killaspy H, Barnes TR et al (2012) Group art therapy as an adjunctive treatment for people with schizophrenia. A randomised controlled trial (MATISSE). Health Technol Assessm (Winchester, England) 16(8): iii–iv, 1–76. https://doi.org/10.3310/hta16080

3. Montag C, Haase L, Seidel D et al (2014) A pilot RCT of psychodynamic group art therapy for patients in acute psychotic episodes. Feasibility, impact on symptoms and mentalising capacity. PloS One 9(11): e112348. https://doi.org/10.1371/journal.pone.0112348

4. Shawyer F, Farhall J, Mackinnon A et al (2012) A randomised controlled trial of acceptance-based cognitive behavioural therapy for command hallucinations in psychotic disorders. Behav Res Ther 50(2):110–121. https://doi.org/10.1016/j.brat.2011.11.007

5. Granholm E, McQuaid JR, McClure FS et al (2005) A randomized, controlled trial of cognitive behavioral social skills training for middle-aged and older outpatients with chronic schizophrenia. Am J Psychiatry 162(3):520–529. https://doi.org/10.1176/appi.ajp.162.3.520

6. Hagen R, Turkington D, Berge T, Gråwe RW (2011) CBT for psychosis: A symptom-based approach. Routledge

7. Klingberg S, Wölwer W, Engel C et al (2011) Negative symptoms of schizophrenia as primary target of cognitive behavioral therapy. Results of the randomized clinical TONES study. Schizophr Bull 37 Suppl 2: S98–110. https://doi.org/10.1093/schbul/sbr073

8. Grant PM, Huh GA, Perivoliotis D et al. (2012) Randomized trial to evaluate the efficacy of cognitive therapy for low-functioning patients with schizophrenia. Arch General Psychiatry 69(2): 121–127. https://doi.org/10.1001/archgenpsychiatry.2011.129

9. Bradshaw W (2000) Integrating cognitive-behavioral psychotherapy for persons with schizophrenia into a psychiatric rehabilitation program. Results of a three year trial. Commun Mental Health J 36(5): 491–500. https://doi.org/10.1023/a:1001911730268

10. Garety PA, Fowler DG, Freeman D et al (2008) Cognitive–behavioural therapy and family intervention for relapse prevention and symptom reduction in psychosis. Randomised controlled trial. The British journal of psychiatry: the journal of mental science 192(6): 412–423. https://doi.org/10.1192/bjp.bp.107.043570

11. Granholm E, Holden J, Link PC et al (2014) Randomized clinical trial of cognitive behavioral social skills training for schizophrenia. Improvement in functioning and experiential negative symptoms. Journal of consulting and clinical psychology 82(6): 1173–1185. https://doi.org/10.1037/a0037098

12. Jenner JA, Nienhuis FJ, Wiersma D et al (2004) Hallucination focused integrative treatment. A randomized controlled trial. Schizophr Bull 30(1): 133–145. https://doi.org/10.1093/oxfordjournals.schbul.a007058

13. Bighelli I, Salanti G, Huhn M et al (2018) Psychological interventions to reduce positive symptoms in schizophrenia. Systematic review and network meta-analysis. World psychiatry: official journal of the World Psychiatric Association (WPA) 17(3): 316–329. https://doi.org/10.1002/wps.20577

14. Roberts DL, Penn DL (2013) Social Cognition in Schizophrenia. Oxford University Press

15. Chadwick P (2006) Person-Based Cognitive Therapy for Distressing Psychosis. John Wiley & Sons Ltd, West Sussex, England

16. Chadwick P, Hughes S, Russell D et al (2009) Mindfulness groups for distressing voices and paranoia. A replication and randomized feasibility trial. Behav Cogn Psychother 37(4): 403–412. https://doi.org/10.1017/S1352465809990166

17. Chien WT, Lee IYM (2013) The mindfulness-based psychoeducation program for Chinese patients with schizophrenia. Psychiatric serv (Washington, D.C.) 64(4):376–379. https://doi.org/10.1176/appi.ps.002092012

18. Martin LAL, Koch SC, Hirjak D et al (2016) Overcoming disembodiment. The effect of movement therapy on negative symptoms in schizophrenia-a multicenter randomized controlled trial. Front Psychol 7:483. https://doi.org/10.3389/fpsyg.2016.00483

19. Talwar N, Crawford MJ, Maratos A et al (2006) Music therapy for in-patients with schizophrenia. Exploratory randomised controlled trial. The British J Psychiatry 189:405–409. https://doi.org/10.1192/bjp.bp.105.015073

20. Schrank B, Brownell T, Jakaite Z et al (2016) Evaluation of a positive psychotherapy group intervention for people with psychosis. Pilot randomised controlled trial. Epidemiol Psychiatric Sci 25(3): 235–246. https://doi.org/10.1017/S2045796015000141

21. Cather C, Penn D, Otto MW et al (2005) A pilot study of functional Cognitive Behavioral Therapy (fCBT) for schizophrenia. Schizophr Res 74(2–3): 201–209. https://doi.org/10.1016/j.schres.2004.05.002

22. Valencia M, Murow E, Rascon ML (2006) Comparación de tres modalidades de intervención en esquizofrenia: terapia psicosocial, musicoterapia y terapias múltiples. [Comparison of three types of treatment for schizophrenia: Psychosocial therapy, music therapy, and multiple therapies]. Revista Latinoamericana de Psicologia 38(3):535–549

23. Haddock G, Barrowclough C, Shaw JJ et al (2009) Cognitive-behavioural therapy v. social activity therapy for people with psychosis and a history of violence. Randomised controlled trial. British J Psychiatry 194(2):152–157. https://doi.org/10.1192/bjp.bp.107.039859

24. Durham RC, Guthrie M, Morton RV et al (2003) Tayside-Fife clinical trial of cognitive-behavioural therapy for medication-resistant psychotic symptoms. Results to 3-month follow-up. British J Psychiatry 182:303–311. https://doi.org/10.1192/bjp.182.4.303

25. Penn DL, Meyer PS, Evans E et al (2009) A randomized controlled trial of group cognitive-behavioral therapy vs. enhanced supportive therapy for auditory hallucinations. Schizophr Res 109(1–3):52–59. https://doi.org/10.1016/j.schres.2008.12.009

26. Pot-Kolder RMCA, Geraets CNW, Veling W et al (2018) Virtual-reality-based cognitive behavioural therapy versus waiting list control for paranoid ideation and social avoidance in patients with psychotic disorders. A single-blind randomised controlled trial. Lancet Psychiatry 5(3):217–226. https://doi.org/10.1016/S2215-0366(18)30053-1

27. Hayes SC, Hofmann SG (2017)The third wave of cognitive behavioral therapy and the rise of process-based care. World Psychiatry16(3):245–246. https://doi.org/10.1002/wps.20442

28. de Jong S, van Donkersgoed RJM, Timmerman M E et al (2019) Metacognitive reflection and insight therapy (MERIT) for patients with schizophrenia. Psychol Med 49(2):303–313. https://doi.org/10.1017/S0033291718000855

29. Gürcan MB, Yildiz M, Patir K, Demir Y (2021) The effects of narrative and movie therapy on the theory of mind and social functioning of patients with schizophrenia. Noro Psikiyatri Arsivi 58(2):108–114. https://doi.org/10.29399/npa.27291

30. Palma C, Farriols N, Frías A, Cañete J, Gomis O, Fernández M, Alonso I, Signo S (2019) Randomized controlled trial of cognitive-motivational therapy program (PIPE) for the initial phase of schizophrenia: Maintenance of efficacy at 5-year follow up.^✰^. Psychiatry Res 273:586–594. https://doi.org/10.1016/j.psychres.2019.01.084

### Risk-of-bias assessment

Six, 25 and 27 studies were judged to be at low, moderate and high overall risk of bias, respectively (Table 2).

**Fig. 2:**
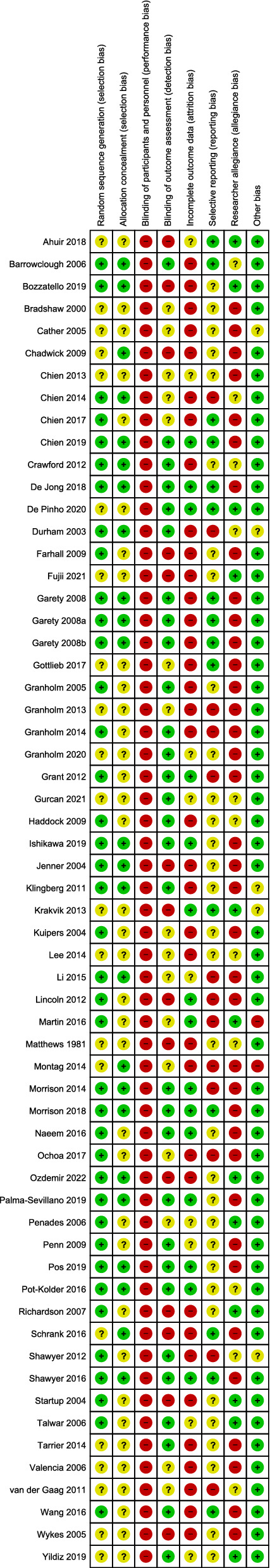
Risk-of-bias judgements for the included studies. Reviewers’ judgements about each risk of bias item for each included study. (Created with Review Manager 5.3)

Concerning random sequence generation, the risk of bias was low in 40 (69%) studies; concerning allocation concealment, it was low in 25 (43%) studies; concerning blinding of participants and personnel, the risk of bias was never low; concerning blinding of outcome assessment in 29 (50%) studies; concerning attrition bias in 15 (26%) studies; concerning selective reporting in 15 (26%) studies; concerning researchers’ allegiance in 12 (21%) studies; and in 53 (91%) studies concerning other bias (Fig. 2).

### All psychological interventions compared to all control conditions (primary analysis)

**Fig. 3:**
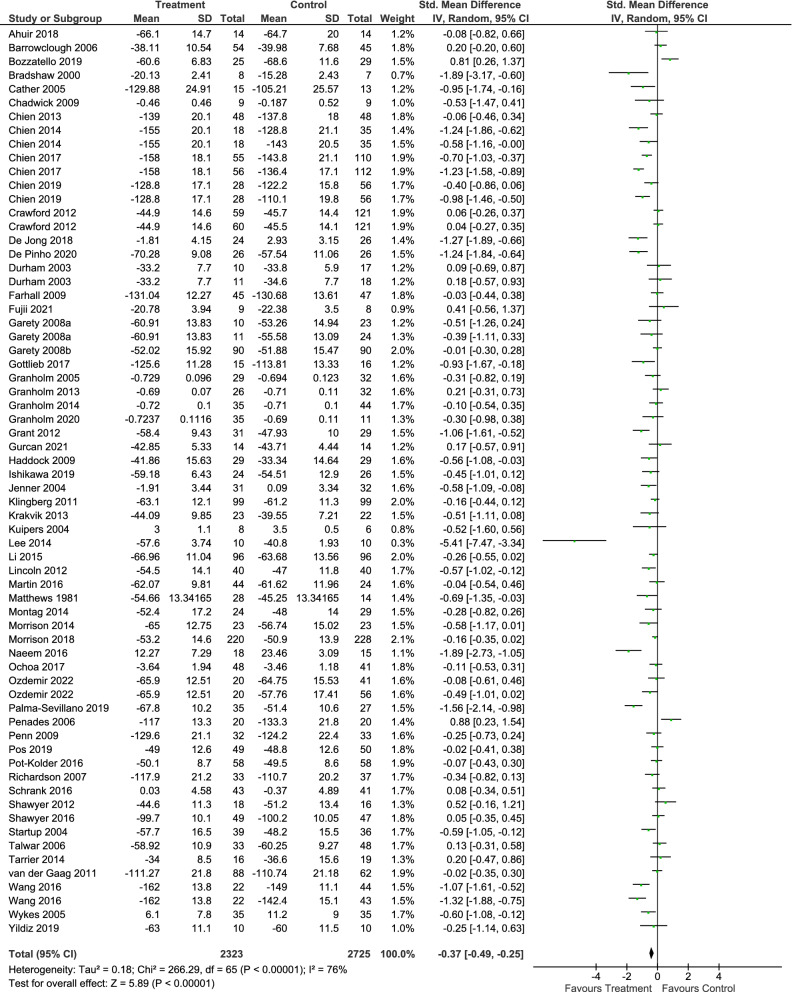
Forest plot all psychological interventions versus control. (Created with Review Manager 5.3)

58 studies with 5048 participants provided data for this analysis. Psychological treatments were associated with a greater improvement in participants’ functioning scores in comparison to control conditions (SMD =  – 0.37, 95% CI  – 0.49 to  – 0.25), with substantial heterogeneity (*I*^2^ = 76%) (Fig. 3). The confidence in the estimate assessed with the GRADE approach was judged to be very low, due to the presence of studies at high risk of bias, substantial heterogeneity and suspected publication bias (Table 4).

**Table 4:** GRADE evidence profile. (Created with GRADEpro)

Certainty assessment							No of patients		Effect		Certainty	Importance
No of studies	Study design	Risk of bias	Inconsistency	Indirectness	Imprecision	Other considerations	Psychological interventions	Control conditions	Relative (95% CI)	Absolute (95% CI)		
*Functioning*												
58	Randomized trials	Serious^a^	Serious^b^	Not serious	Not serious	Publication bias strongly suspected^c^	2323	2725	–	SMD **0.37 SD lower** (0.49 lower to 0.25 lower)	⨁◯◯◯ Very low	

Statistically significance values are in bold

*CI* confidence interval, *SMD* standardized mean difference

Explanations

^a^The proportion of information from studies at high risk of bias is sufficient to affect the interpretation of results

^b^I-squared = 76%

^c^Visual inspection of funnel plot suggests some asymmetry, confirmed by Egger's test (*p* = 0.0097)

### Groups of psychological interventions and specific psychological interventions compared to control conditions

#### CBT versus control

**Fig. 4:**
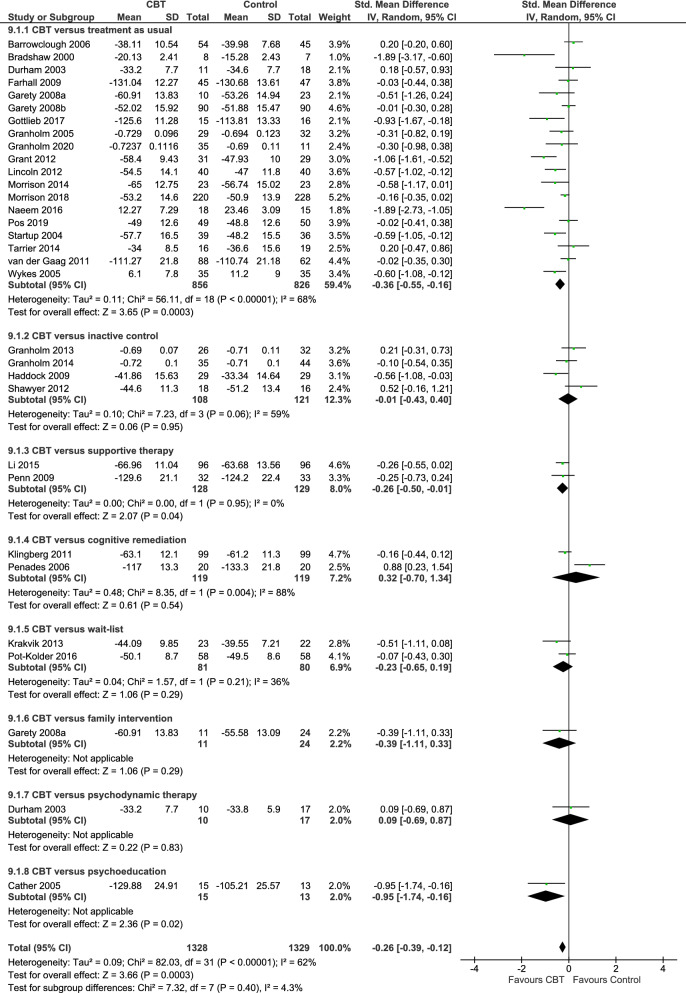
Forest plot CBT versus control. (Created with Review Manager 5.3)

30 studies with 2657 participants provided data for this analysis. Overall, CBT was associated with a greater improvement in functioning (SMD =  – 0.26, 95% CI  – 0.39 to  – 0.12), with substantial heterogeneity (*I*^2^ = 62%) (Fig. 4). The benefit was clear in comparison with TAU (SMD =  – 0.36, 95% CI  – 0.55 to  – 0.16), supportive therapy (SMD =  – 0.26, 95% CI  – 0.50 to  – 0.01) and psychoeducation (SMD =  – 0.95, 95% CI  – 1.74 to  – 0.16), while for the comparisons with inactive control, cognitive remediation, wait-list, family intervention and psychodynamic therapy, the confidence intervals include the possibility of no difference.

#### Third-wave cognitive behavior therapies versus control

**Fig. 5:**
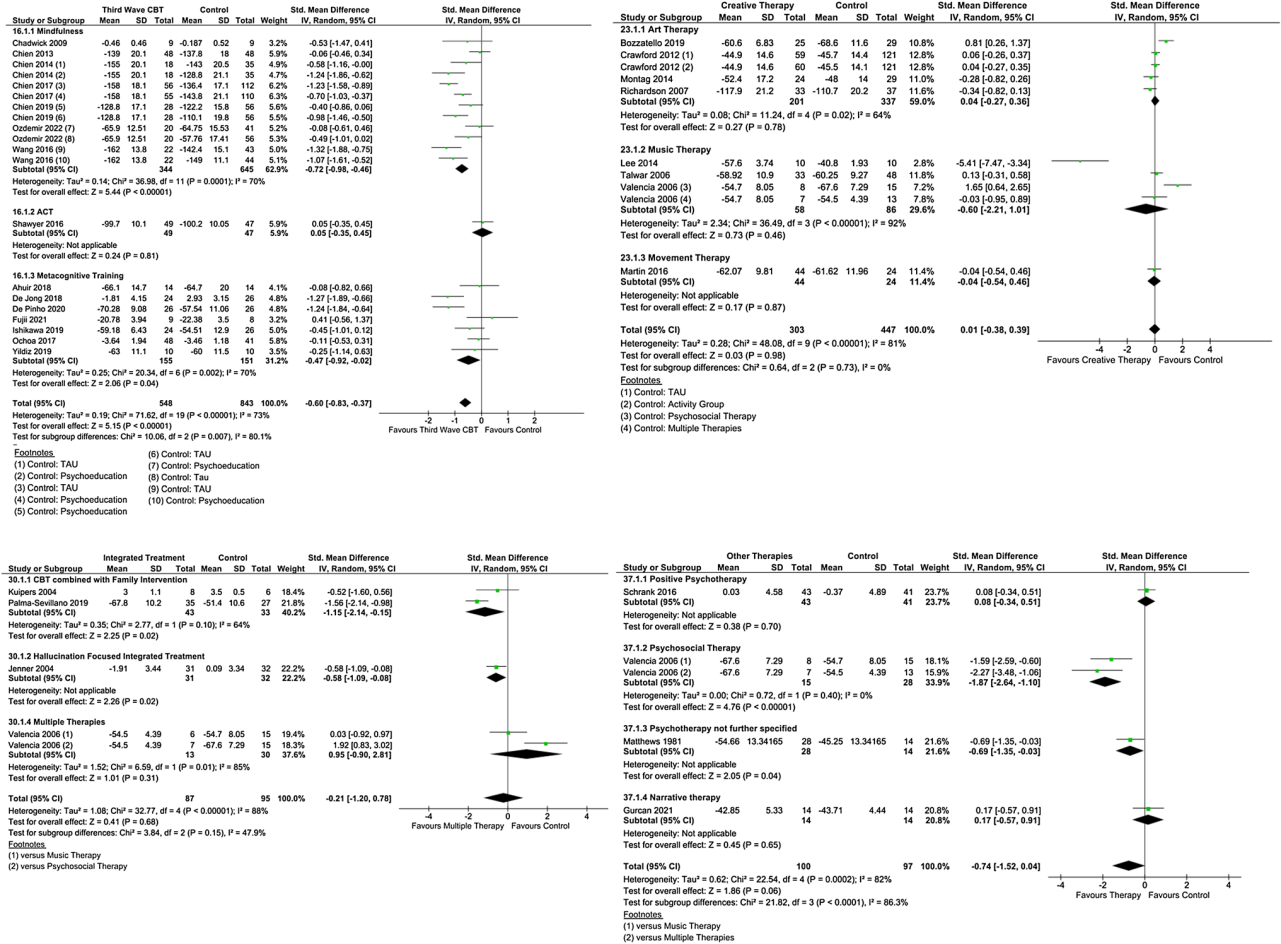
Third-wave, creative, multiple and other therapies versus control. (Created with Review Manager 5.3 and Microsoft Power Point)

15 studies with 1391 participants were included in this analysis. Third-wave CBT interventions were associated with an improvement in functioning (SMD =  – 0.60, 95% CI  – 0.83 to  – 0.37), with substantial heterogeneity (*I*^2^ = 73%) (Fig. 5).

Of these, seven studies investigated mindfulness (SMD =  – 0.72, 95% CI  – 0.98 to  – 0.46), one study investigated ACT (SMD = 0.05, 95% CI  – 0.35 to 0.45) and seven studies investigated metacognitive training (SMD =  – 0.47, 95% CI  – 0.92 to  – 0.02).

#### Creative therapies versus control

In this analysis, eight studies on art therapy, music therapy and movement therapy provided data for 750 participants. No difference was found between creative therapies and the control group (SMD = 0.01, 95% CI  – 0.38 to 0.39), with considerable heterogeneity (*I*^2^ = 81) (Fig. 5).

Four studies investigated art therapy (SMD =  – 0.04, 95% CI  – 0.27 to 0.36), three studies music therapy (SMD =  – 0.60, 95% CI  – 2.21 to 1.01) and one study movement therapy (SMD =  – 0.04, 95% CI  – 0.54 to 0.46).

#### Integrated therapies versus control

Four studies with 182 participants were included for this comparison. No difference between integrated therapies and control group was found (SMD =  – 0.21, 95% CI  – 1.20 to 0.78), with considerable heterogeneity (*I*^2^ = 88%) (Fig. 5).

Two studies investigated CBT combined with family intervention (SMD =  – 1.15, 95% CI  – 2.14 to  – 0.15), one study hallucination focused integrated treatment (SMD = 0.58, 95% CI  – 1.09 to  – 0.08) and one study multiple therapies (SMD = 0.95, 95% CI  – 0.90 to 2.81).

#### Other therapies versus control

Among other therapies, we included positive psychotherapy, psychosocial therapy, narrative therapy and not further specified psychotherapy. Based on four studies with 197 participants, these interventions were not associated with an improvement in functioning (SMD =  – 0.74, 95% CI  – 1.52 to 0.04), with considerable heterogeneity (*I*^2^ = 82%) (Fig. 5).

One study investigated positive psychotherapy (SMD = 0.08, 95% CI  – 0.34 to 0.51), one study investigated psychosocial therapy (SMD =  – 1.87, 95% CI  – 2.64 to  – 1.10), one study psychotherapy, without further specification (SMD = -0.69, 95% CI  – 1.35 to  – 0.03), and one study narrative therapy (SMD = 0.17, 95% CI  – 0.57 to 0.91).

### Subgroup analyses

#### Treatment setting: individual versus group

In 28 studies, the psychological intervention was delivered in a group setting (SMD =  – 0.38, 95% CI  – 0.57 to  – 0.20, *I*^2^ = 78%) and in 25 studies in an individual setting (SMD =  – 0.31, 95% CI  – 0.48 to  – 0.14, *I*^2^ = 70%).

Test for subgroup difference did not find a difference between these two subgroups (*p* = 0.56).

#### Therapist expertise: trainee therapist allowed vs only expert therapists

In 32 studies, only expert therapists conducted therapy (SMD =  – 0.39, 95% CI  – 0.55 to  – 0.22, *I*^2^ = 79%), in seven studies’ therapists in training conducted treatment, as well (SMD =  – 0.16, 95% CI  – 0.34 to 0.03, *I*^2^ = 17%). Test for subgroup difference did not find a difference between these two subgroups (*p* = 0.07).

### Metaregression analyses

The effect of psychological interventions on functioning was not found to be associated with *number of sessions* (*p* = 0.4347), s*tudy duration* (*p* = 0.0901), *male percentage* (*p* = 0.1636), *or baseline severity* (*p* = 0.1244).

*Age* was found to have a role in moderating treatment effect on functioning, with a possible bigger treatment effect for younger patients (*p* = 0.0072) (Table 5).

**Table 5:** Results of meta-regression analyses. (Adapted from [1], created with Microsoft Office)

Moderator	Coefficient	95% CI	Z value	*P* value
Number of sessions	0.0047	– 0.0071; 0.0164	0.7811	0.4347
Study duration	– 0.0063	– 0.0136; 0.0010	– 1.6950	0.0901
Mean age	0.0222	0.0060; 0.0383	2.6892	**0.0072**
Male percentage	0.0077	– 0.0031; 0.0184	1.3930	0.1636
Baseline severity	– 0.0080	– 0.0182; 0.0022	– 1.5364	0.1244

Satistically significant values are in bold (*p* < 0.05)

### Sensitivity analyses

Excluding 14 *open label* studies did not substantially change the results of the analysis (SMD =   –  0.38, CI  – 0.52 to  – 0.25). Heterogeneity remained similar to the original analysis (*I*^2^ = 77%).

Excluding 34 studies with *high researcher allegiance*, the confidence interval includes the possibility of no difference between the psychological interventions and the control condition (SMD =   –  0.21, CI  – 0.42 to 0.00). Heterogeneity remained similar compared to the original analysis (*I*^2^ = 75%).

Excluding 27 studies with *high overall risk of bias* did not change the results of the analysis substantially (SMD =   –  0.44, CI  – 0.58 to  – 0.25). Heterogeneity remained similar (I^2^ = 79%).

Excluding 11 studies focused on *treatment resistant patients* led to a slight decrease of effect size (SMD =   – 0.42, CI  – 0.60 to  – 0.27). Heterogeneity remained similar (*I*^2^ = 80%).

### Publication bias

**Fig. 6:**
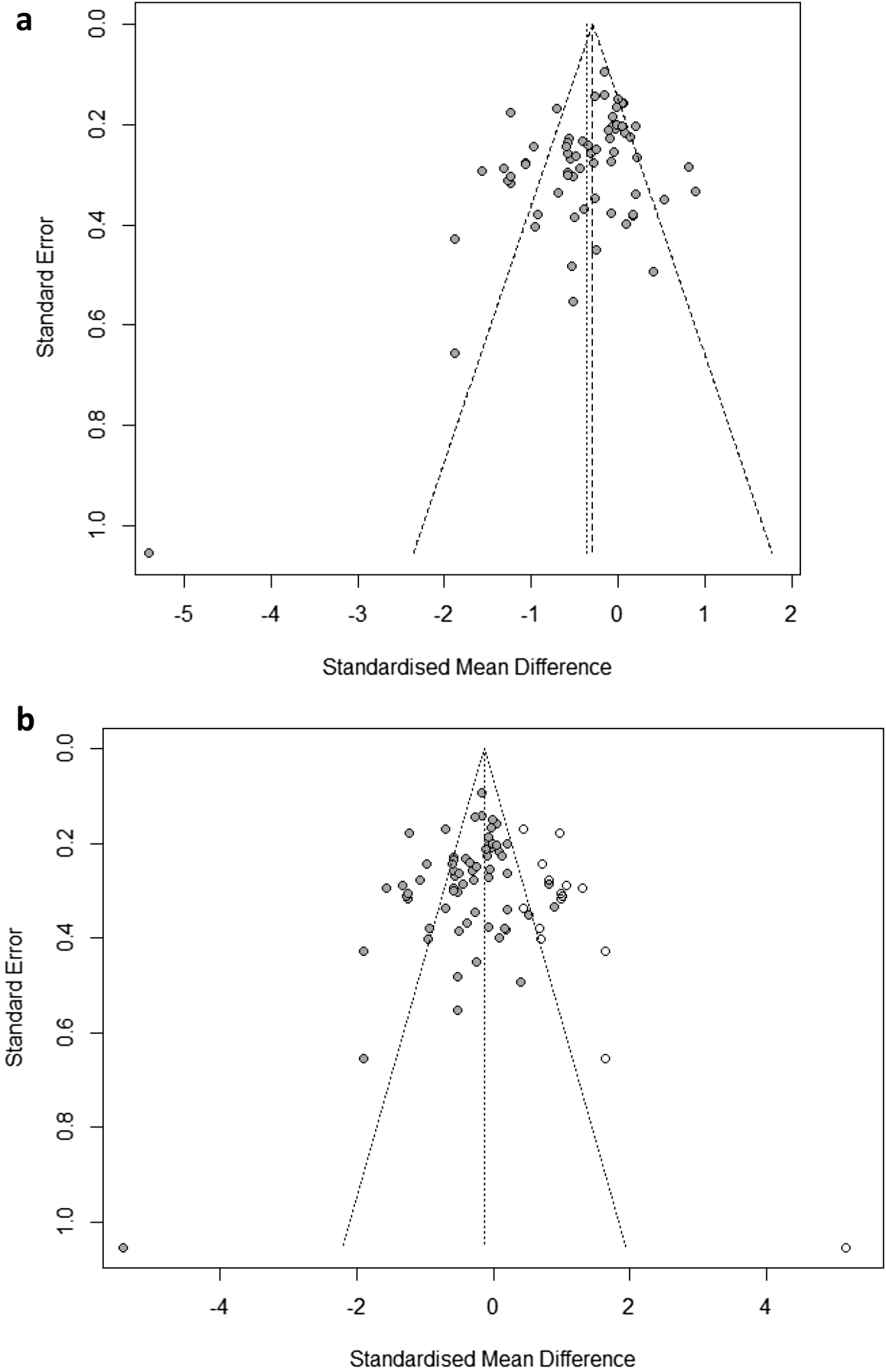
Funnel plot. (Created with R Studio version 1.3.959 and Microsoft Power Point): **a** shows the funnel plot for the comparison all psychological interventions versus all control conditions. In **b**, the trim-and-fill method by Duval and Tweedie was applied; 16 studies added are represented as white dots

Visual inspection of the funnel plot reveals some asymmetry, suggesting that small studies favoring the control condition could have remained unpublished (Fig. 6a). Egger’s test for funnel plot asymmetry confirmed this (*p* = 0.0097) [36]. By applying the trim-and-fill method by Duval and Tweedie 16 studies were added to the funnel plot (represented in white in Fig. 6b) confidence intervals included the possibility of no effect (SMD =  – 0.13, 95% CI  – 0.27 to 0.01) [37].

## Discussion

### Summary of main results

We conducted the first systematic review and meta-analysis investigating the effect of psychological interventions for functioning in patients with schizophrenia. After a thorough literature search, we were able to include 58 studies with usable outcome data.

We found that psychological interventions overall showed a benefit in improving functioning compared to control conditions. According to Cohen (0.2 = small, 0.5 = medium, 0.8 = large effect size), this effect size can be considered small to medium [34]. CBT compared to any control condition had also a small effect in improving functioning, third-wave CBT interventions produced a medium benefit. Creative therapies, integrated therapies and other therapies were not associated with an improvement in functioning.

### Discussion in light of previous literature

In a previous network meta-analysis by our group focused on acute patients with positive symptoms, we found an effect of  – 0.25 (95% CI  – 0.48 to  – 0.03) indicating a benefit for CBT compared to treatment as usual [14], while in the present work on the general population of patients with schizophrenia, the effect of CBT vs TAU was larger ( – 0.43). It can be argued that, after acute symptoms of schizophrenia are treated, patients are more receptive for interventions aimed at improving functioning. Results of Bighelli et al. on CBT compared with supportive therapy, family intervention, inactive control and wait-list are in line with the ones of the present work, not showing a difference between these interventions [14]. It must be noted, however, that evidence for these interventions is based on only 1–2 studies each.

Our results are also in line with the findings of Laws et al. that found an effect of 0.25 (95% CI 0.10–0.39) showing a benefit for CBT compared with control conditions [15].

Contradicting the findings of the present meta-analysis, Jones found no evidence supporting the use of CBT for functioning in people with schizophrenia [10]. This conclusion is, however, strongly affected by the different analysis that the authors conducted, analyzing different rating scales and different time points separately, so that our results cannot be compared with the ones of the Cochrane review.

In the present analysis, findings on mindfulness, metacognitive training, hallucination focused integrated treatment and psychosocial therapy are promising, but based only on a small number of trials. A recent review focused on metacognitive training, including randomized and non-randomized evidence, found a similar effect on functioning (SMD 0.41, 95% CI 0.12 to 0–69) [96].

Results of subgroup, meta-regression and sensitivity analyses did not find a role for the investigated variables in moderating the effect of psychological interventions on functioning, with exception of a possible moderating role for age (with bigger treatment effects associated with younger patients) and for researchers’ allegiance.

Younger patients might be more open for change and to engage in a psychotherapy. A systematic review reported larger effect sizes for psychotherapy in young adults with depression (up to 24 years) than in older adults [97].

Excluding studies that were conducted by the same authors who developed the treatment manual, the effect of the interventions on functioning was not so clear anymore, suggesting that effects might be inflated by allegiance of the authors to the investigated interventions. It must be noted that heterogeneity remained high in all subgroup analysis, confirming that the variables investigated in the subgroup analyses did not account for heterogeneity. A possible further explanation for heterogeneity in the investigated studies could be the use of different control conditions, that we pooled together. A network meta-analysis approach could help to disentangle this issue, analyzing also control conditions as different nodes of the network.

### Limitations

First, the pooling of different rating scales is a problematic issue. As the concept of functioning developed through the years, the rating scales changed as well, including and giving a focus on different aspects like living skills, disability, social and occupational role [1, 6]. Moreover, some scales, such as the Global Assessment Scale (GAS), include psychopathology and some do not, for example the Social and Occupational Functioning Assessment Scale (SOFAS) [98, 99]. To account for this difficulty, we decided to include only published scales, for which it is possible to check the original reference and therefore description and metric properties, and we applied a statistical correction, by calculating SMDs. Still, many of the comparisons present a considerable heterogeneity, and one of the possible causes could be the use of different rating scales. On the contrary, the strategy in Cochrane reviews is to keep every measure separate, but paying the price of losing the overall picture [10, 16]. An ideal solution does not exist; an agreement on one functioning measure would make research results more comparable. Currently, in the Diagnostic and Statistical Manual 5, the American Psychiatric Association suggests using the World Health Organization Disability Assessment Schedule (WHODAS) 2.0. [100]

Second, pooling and classifying psychological treatments are not straightforward. We adopted a transparent approach, classifying the interventions according to the description given in each study, and presenting the assumptions made in Table 3. We also present different level of analyses for treatment grouped and taken singularly, so that an appraisal of the evidence is provided also independently from our classification of the psychological interventions.

Third, of 253 studies that met our eligibility criteria, only 58 reported data on functioning as an outcome. Most of all, there was scarcity of evidence for interventions other than CBT, and for some interventions, the evidence is based on few studies only. Results need therefore to be interpreted with caution.

A further limitation of the current analysis, and of studies on psychological interventions in patients with schizophrenia in general, is that participants of the studies are generally also receiving antipsychotic medication. Insufficient details on the medication were provided in the studies, so that it was not possible to disentangle the effect of psychological and pharmacological therapy. Randomization ensures that the observed effect sizes refer to the presence of the psychological intervention.

Finally, the certainty of the evidence was evaluated as very low with the GRADE approach. This evaluation is motivated by three aspects; (i) the studies providing data are mostly at overall moderate or high risk of bias; (ii) there was substantial heterogeneity. This may be due to the fact that we analyzed the results of studies with different duration together. The meta-regression analysis investigating the role of study duration was of borderline significance, so this aspect remains unclear; (iii) the results are potentially affected by small study effect, that can be associated with publication bias. Even if we conducted a thorough literature search, including study registries and gray literature, it is possible that some small studies favoring the control condition remained unpublished and were not possible to detect.

### Implications for future research and practice

Despite limitations, the present data suggest that psychological interventions can improve functioning in people with schizophrenia. In particular, CBT and third-wave CBT interventions seem to have a positive effect on functioning.

To increase the amount of evidence on other treatments, future trials investigating psychological interventions for schizophrenia should address functioning among outcomes, not only psychopathology.

## Data Availability

The datasets used and/or analyzed during the current study are available from the corresponding author on reasonable request.

## Code availability

R codes used for analysis of the present work are available from the corresponding author on reasonable request.
